# A numerical study towards shape memory alloys application in orthotic management of pediatric knee lateral deviations

**DOI:** 10.1038/s41598-023-29254-z

**Published:** 2023-02-06

**Authors:** M. G. Alonso, A. Yawny, G. Bertolino

**Affiliations:** 1 División Física de Metales, CNEA, 8400 Bariloche, Argentina; 2 Instituto Balseiro, Universidad Nacional de Cuyo, Bariloche, Argentina; 3 CONICET, Patagonia Norte, 8400 Bariloche, Argentina

**Keywords:** Bioinformatics, Paediatric research, Biomedical engineering

## Abstract

Exerting a constant load would likely improve orthosis effectiveness in treating knee lateral deviations during childhood and early adolescence. Shape memory alloys are potential candidates for such applications due to their so called pseudoelastic effect. The present study aims to quantitatively define the applicable mechanical loads, in order to reduce treatment duration while avoiding tissular damage and patient discomfort. This is essential for performing a more efficient design of correction devices. We use a patient-specific finite elements model of a pediatric knee to determine safe loading levels. The achievable correction rates are estimated using a stochastic three-dimensional growth model. Results are compared against those obtained for a mechanical stimulus decreasing in proportion to the achieved correction, emulating the behavior of conventional orthoses. A constant flexor moment of 1.1 Nm is estimated to change femorotibial angle at a rate of (7*.*4 ± 4*.*6) deg/year (mean ± std). This rate is similar to the achieved by more invasive growth modulation methods, and represents an improvement in the order of 25% in the necessary time for reducing deformities of (10 ± 5) deg by half, as compared with conventional orthoses.

## Introduction

Treatment of some childhood deformities is based on the concept of altering growth by the application of mechanical loads^1^. Angular deformities of the knee are often reduced with staples that are placed across the epiphyseal plate (also called growth plate or physis in what follows) in such a manner that further growth increases the pressure on the inner surfaces of the device blades^2^. This slows growth in the device proximal zones, allowing to correct the angular deviation of the treated bones^3^. The technique is only effective for treating femorotibial angle deviations between 7° and 14°, making more invasive approaches necessary for patients near growth stop or suffering deviations larger than 14°^1,4,5^. For femorotibial angle deviations smaller than 7°, the use of night-time orthoses is recommended, although in some cases it does not suffice to avoid a surgical intervention, making orthotic management of such pathologies a controversial approach^4,6^. The lack of standardization of the used devices, the difficulty in regulating the treatment regime, the lack of rigorous clinical trials having adequately monitored control groups and the lack of modern studies in the topic have recently been recognized as the main reasons for this^7^.

Traditionally, orthoses fabrication takes a personalized, rather heuristic approach, often based in non quantitative premises as the Hueter–Volkman law^8^. The forces exerted by these devices are calibrated by the orthopedist, mostly depending on the comfort degree of the patient. Common designs rely in the use of thermoplastic sheets shaped in such a manner that forces tending to correct the existing deviation arise due to the material deformation required to fasten the device on the patient^7^. A classical configuration of knee orthoses consists of metallic and nearly rigid bars that are adjusted onto the patient’s leg by elastic strands^9^. The correction force is regulated by adjusting the length of the elastic strands, or by modifying the device geometry in the case of thermoplastic orthoses.

In the typical geometries used for orthoses construction, using linear elastic materials naturally causes the forces exerted by these devices on the patient to decrease as correction proceeds. Also, the use of elastic strands for its fixation makes it difficult to accurately control the applied force, which is consequently determined by how tight the patient care-taker secures the orthosis each time.

All the previously mentioned factors attempt against the effectiveness of the orthopedic treatment. One question that naturally arises here is whether a device able to guarantee the application of a constant load, previously determined as mechanobiologically adequate, might represent a substantive improvement in treating this type of growth pathologies. In this sense, the incorporation of shape memory alloys (SMAs) as load bearing components in orthoses represents an interesting alternative. In effect, for example under uniaxial loading, alloys such as NiTi are known for their capacity of sustaining around 10% deformation, under a constant load, in reversible manner^10,11^. This behavior allows to devise elements acting as constant-force springs, and for this reason NiTi has been used in a variety of medical and engineering applications^12^. Among these, it is worth to mention braces commonly used for correcting teeth deviations in orthodontics, as they have a somewhat similar goal. In these devices, the pseudoelastic effect is used to assert that the force exerted on the teeth does not decrease as treatment proceeds, avoiding the necessity of frequent re-adjustments existing with formerly used steel made apparatuses^13^. In the field of orthopedics, shape memory alloys have been used as passive actuators for knee orthoses^14^, in ankle foot orthoses for persons having drop foot syndrome^15^, as active elements inserted into textiles^16^ and as deformable elements in orthoses for patients with spastic knee^17^.

It is worthwhile to mention here that the development of devices for the treatment of growth pathologies markedly differs from the fore listed cases, in which the intended effects are immediately measurable once the patient starts wearing the orthosis. In the case of growth pathologies, orthoses efficacy is typically assessed after treatment periods of around 30 months^6^. This makes the precise quantification of applied forces effects to be of upmost importance for their design^18^. The mechanical loads exerted by any orthotic device should be as large as possible to reduce the treatment duration, but at the same time, should not injury or excessively discomfort the patient. However, there is a lack of appropriate information in the literature about these important points^7^.

In this study, we analyze stress and growth in the pediatric knee to clarify these matters, by using a patient-specific finite elements model of this joint. Two main goals are pursued: to bound applicable load levels in order to avoid damage in the epiphyseal plate, as well as in the knee articular tissues, and to estimate the treatment effectiveness, allowing to ponder possible alternatives in the patient’s best interest. For this, stresses in the epiphyseal plates were computed, and a criterion for limiting the effort to be generated by a hypothetical orthosis was established based on existing data obtained from epiphysiolisis treatments^19^. Stresses in the articular tissues were also computed for typical physiological loads occurring while standing, walking, running and cycling. The obtained mechanical solicitations in the knee tissues were compared against the ones produced by an orthotic device for correcting *genu varum*, to bound the applicable effort based on several criteria. The obtained loads were then used to estimate the achievable correction rates, both in the femur and the tibia anatomical lateral deviation angles. For this, we introduced a novel endochondral growth model of the epiphyseal plate into a recently developed finite elements framework^20,21^. Albeit the modeled phenomena are naturally subjected to stochastic variations, modeling should be focused towards having reasonably good estimations of the most probable outcome for the orthotic treatment. To achieve this, a MonteCarlo approach was used, by computing growth in a fairly wide range of possible model parameters. A statistical analysis of predicted outcomes was made. Aspects related to code verification, validation and uncertainty quantification of obtained solutions were specially emphasized^22^.

## Methods

**Figure 1:**
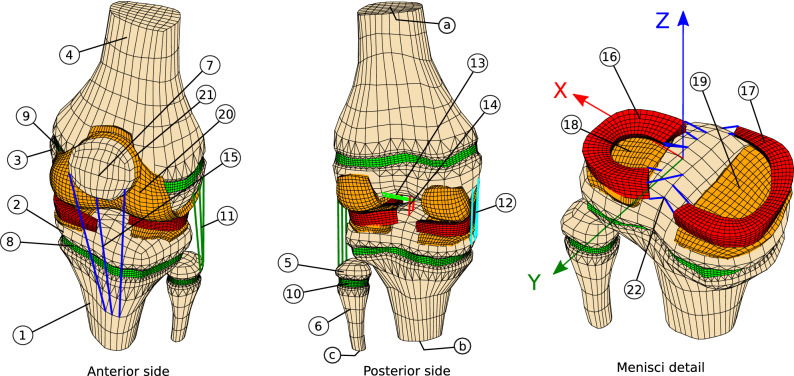
Finite elements model used in the study. Epiphyseal plates have been colored in green. Geometry was obtained from a left 10 years old female patient MRI. (1) tibia diaphysis (2) tibia epiphysis (3) femur epiphysis (4) femur diaphysis (5) fibula epiphysis (6) fibula diaphysis (7) patella (8) tibia physis (9) femur physis (10) fibula physis (11) lat. colat. ligament (12) med. colat. ligament (13) ant. cruc. ligament (14) post. cruc. ligament(15) patellar tendon (16) lat. meniscus (17) medial meniscus (18) lat. tibial cartilage (19) med. tibial cartilage (20) fem. art. cartilage (21) pat. art. cartilage (22) menisci horns.

Data from a nuclear MRI scanning of a female 10 year old patient left knee, 45 kg body weight (BW) and 1.5 m height were segmented using 3D slicer^23,24^. Data was obtained with the informed consent of the patient legal guardians, in accordance with all relevant guidelines and regulations. Use of data for the present study was approved by the Fundación Intecnus ethics committee. An initial femorotibial angle deviation of (4 ± 1)° was measured. Segmented geometry was post-processed with Blender^25^ and Salome Mesh^26^ so as to obtain a regular, hexahedral finite elements mesh, Fig. 1. For each modeling case, mesh size was prescribed after a mesh convergence analysis, for which initial elements in the interest region were subsequently split in half, and Richardson extrapolation was used for the estimation of the numerical solution global error^27^ (Sup. Mats. 1, 2). Maximum geometry local approximation error was estimated to be in the order of 0.5 mm.

Model geometry is subdivided in 22 sub-regions, as detailed in Fig. 1. In the following, and unless specified, mechanical properties were set as particularized in Table 1. Reference frame for subsequent computations was defined as a positively oriented triplet, being the *z* axis coincident with the mechanical axis of the limb and the *xz* plane parallel to the patient coronal plane. Finite elements computations were performed with Cast3M^28^.

**Table 1:** Considered anatomical segments and mechanical properties of the used FE model.

Segment	Denomination	Mechanical behavior	Mechanical properties	References
1–7	Bony regions	Rigid solids	–	^29–31^
8–10	Physes	Linear elastic solid	$\mathrm{E}=12\mathrm{ MPa},\upnu =0.45$	^3^
11	Lat. colat. ligament	Non linear spring	$\upkappa =3.4\frac{\mathrm{kN}}{\mathrm{mm}},{\upvarepsilon }_{1}=0.01,{\upvarepsilon }_{0}=0.04$	^21,29–31^
12	Med. colat. ligament		$\upkappa =4.5\frac{\mathrm{kN}}{\mathrm{mm}},{\upvarepsilon }_{1}=0.03,{\upvarepsilon }_{0}=0.04$	
13	Ant. cruc. ligament		$\upkappa =5.6\frac{\mathrm{kN}}{\mathrm{mm}},{\upvarepsilon }_{1}=0.03,{\upvarepsilon }_{0}=0.1$	
14	Post. cruc. ligament		$\upkappa =10.1\frac{\mathrm{kN}}{\mathrm{mm}},{\upvarepsilon }_{1}=0.03,{\upvarepsilon }_{0}=-0.25$	
15	Patellar tendon		$\upkappa =22.5\frac{\mathrm{kN}}{\mathrm{mm}},{\upvarepsilon }_{1}=0.01,{\upvarepsilon }_{0}=0.00$	
16,17	Menisci	Linear elastic solid	$\mathrm{E}=59\mathrm{ MPa},\upnu =0.45$	
18–21	Articular cartilages		$\mathrm{E}=20\mathrm{ MPa},\upnu =0.45$	
22	Menisci horns	Rigid link	–	^29,31^

Reference numbers as detailed in Fig. 1.

### Growth plate stress

Stresses arising in the physes caused by efforts exerted on the bones diaphyses were considered to be independent of the elastic behavior of articular tissues, as can be derived from the free body analysis of Sup. Fig. 1. Consequently, a reduced model representing only the epiphyses, the physes and the diaphyses of the femur and the tibia was considered for obtaining stresses on the epiphyseal plate. Physis material behavior was modeled as linear elastic^32,33^. The orthotic action was modeled as equivalent to two couples originating in forces acting in the femur and the tibia (Sup. Fig. 1). This couples are afterwards considered as two moments of 1 Nmm parallel to the *y* direction, acting at the barycenters of surfaces a and b, Fig. 1. Displacements were fixed in the epiphyseal zones (2 and 3, Fig. 1). The scripts that have been used for these evaluations are provided to guarantee the study reproducibility (Sup. Mat. 3). After a convergence analysis, global error in computed stresses was estimated to be less than 2% (Sup. Fig. 3). Converged mesh in this case consists of 6440 linear tetrahedrons, 12869 linear hexahedrons and 8050 linear pyramids (Sup. Mat. 1).

Solution uncertainty was assessed by performing 100 computations of the maximum applicable flexor moment, considering the physis as a linear elastic, nearly incompressible material with a Gaussian Young Modulus distribution of (12*.*0 ± 4*.*3) MPa (mean ± std) and the bone as a linear elastic material with a Gaussian Young Modulus distribution of (15 ± 5) GPa (mean ± std) and a Poisson ratio of 0.3 ± 0.1 (mean ± std)^3^. Three stress intensity estimators were evaluated in the epiphyseal plates, namely, the von Mises, the Tresca and the maximum principal stresses (${\upsigma }_{\mathrm{VM}}$, ${\upsigma }_{\mathrm{T}}$ and ${\upsigma }_{\mathrm{P}}$ respectively). These estimators were limited so as to not surpass an equivalent uniaxial stress level of ${\upsigma }_{\mathrm{adm}}=0.153\mathrm{ MPa}$, that has been found to produce epiphysiolisis under sustained loading in clinical studies^19^. For this, the maximum applicable flexor moment was computed as ${\mathrm{T}}_{\mathrm{max}}=\mathrm{min}\left\{\frac{{\upsigma }_{\mathrm{adm}}}{\mathrm{max}({\upsigma }_{\mathrm{VM}})},\frac{{\upsigma }_{\mathrm{adm}}}{\mathrm{max}({\upsigma }_{\mathrm{T}})},\frac{{\upsigma }_{\mathrm{adm}}}{\mathrm{max}({\upsigma }_{\mathrm{P}})}\right\}\times 1\mathrm{ Nmm }.$

### Stress on the articular tissues

For quantifying mechanical solicitations on the articular tissues, bony regions were modeled as rigid bodies^29,30^. Menisci and articular cartilages were considered as linear elastic, nearly incompressible materials^21,29–31^. Patellar tendon, as well as collateral and cruciate ligaments were modeled as one dimensional, nonlinear spring bundles^31^. The spring axial force f was computed as a function of the ligament strain $\varepsilon$ and two constitutive parameters $\kappa$ and ${\varepsilon }_{1}$. Further details regarding this methodology have been published in a previous work^21^.

In order to bound applicable loads to reduce injury risk on the articular tissues, deformations and stresses were computed for loads representing those arising during typical day to day activities. Then, equivalences were established with stresses and deformations originated by wearing an orthosis. Maximum von Mises (VM) stress in the tissues, peak contact stress in the articular surfaces and maximum deflection of the menisci were considered to be the relevant injury risk estimators. A given orthosis related load was deemed equivalent to a particular activity when the maximum of any of the computed estimators was the same in both conditions, for any of the knee segments. Modeling was carried on by defining a frictionless, non-interpenetrating contact constraint between the menisci and the articular cartilages. An explicit integration algorithm was used for solving the resulting contact problem. Displacements were fixed for the tibia and the fibula distal sections (b and c, Fig. 1). A force parallel to the mechanical axis of the limb was applied at the barycenter of section a, Fig. 1, representing load levels occurring while standing (0.5BW), walking (2.8BW), cycling (1.25BW) and jogging (4.8BW)^34^. Contact stresses, von Mises stresses and menisci deflections were computed for each case. Results were compared against the obtained when the applied load was a moment parallel to the y direction at the barycenter of section a, Fig. 1, representing the mechanical action of an external orthosis. The Cast3M scripts used in this case are provided to ensure reproducibility (Sup. Mat. 4). Converged mesh used for this case consists of 9594 hexahedrons, of which 2887 are linear and 6707 are second order elements (Sup. Mat. 2). Contact mesh consists of 6432 linear triangles.

Uncertainty quantification for this case was considered beyond the scope of the present study, because of two reasons. The first is that loading levels were defined based in a one-to-one comparison between different loading conditions of the same knee model. The second, that equivalent loads were found to be considerably larger than the limiting loads obtained by other criteria, making a more precise estimation of these values irrelevant for the subsequent analyses.

### Growth modeling

Growth in the epiphyseal plate was computed with a phenomenological model incorporating changes in growth speed, direction and ossification rate of the physis tissue^20^. According with this model, growth is described by a deformation rate tensor, defined as:

1 $${\dot{\mathbf{\varepsilon }}}\left( {{\mathbf{x}},{\text{t}}} \right) = {\dot{\mathbf{\varepsilon }}}^{0} \left( {{\mathbf{x}},{\text{t}}} \right) + {\dot{\varepsilon }}_{{\text{B}}} {{\varvec{\upmu}}}\left( {{\mathbf{x}},{\text{t}}} \right)$$

In Eq. 1, ${\dot{\upvarepsilon}} _{{\text{B}}}$ is a factor representing the baseline growth rate of the epiphyseal chondrocytes. The growth speed tensor is determined by the contribution of the initial growth tensor ${\dot{{\varvec{\upvarepsilon}}}}^{0}\left(\mathbf{x},\mathrm{t}\right)$ and a tensor ${\varvec{\upmu}}$ accounting for the histological structure distortion. Tensor ${\dot{{\varvec{\upvarepsilon}}}}^{0}\left(\mathbf{x},\mathrm{t}\right)$ was assumed to be constant in the considered time period. It was determined by solving an inverse problem such that displacements of surfaces a and b are bounded to the z axis, Fig. 1, and corresponds with the predefined growth speeds ${\dot{\mathrm{L}}}_{\mathrm{fem}}$ and ${\dot{\mathrm{L}}}_{\mathrm{tib}}$ of the femur and the tibia when no mechanical loads are applied. These speeds were assumed to be linearly correlated, representative of a normal 10 years old female and following a Gaussian probability distribution^35^, Table 2. The factor ${\dot{\upvarepsilon }}_{\mathrm{B}}$ was computed as $\mathrm{tr}({\dot{{\varvec{\upvarepsilon}}}}_{0})$.

**Table 2:** Growth model parameters values.

Parameter	Mean value	Std
${\upkappa }_{1}{\uptau }_{1}$	− 1.5 MPa^−1^ day	0.75 MPa^−1^ day
${\upgamma }_{0}$	3.5 MPa^−1^	1.75 MPa^−1^
${\upgamma }_{1}$	5.5${\upgamma }_{0}$	
${\uptau }_{1}$	1 day	0.5 day
${\upnu }_{1}$	2.6 day	1.3 day
${\mathrm{E}}_{\mathrm{f}}$	12 MPa	4.3 MPa
θ_0_	10°	5°
${\uplambda }_{\mathrm{use}}$	10.5 h	1.5 h
${\dot{\mathrm{L}}}_{\mathrm{fem}}$	1.49 cm/year	0.24 cm/year
${\dot{\mathrm{L}}}_{\mathrm{tib}}$	$0.857 {\dot{\mathrm{L}}}_{\mathrm{fem}}$	

Unless indicated, parameters were considered to be non correlated, and behaving according to a Gaussian probability distribution.

The distortion term ${{\varvec{\upmu}}}\left( {{\mathbf{x}},{\text{t}}} \right){ }$ is obtained as a function of applied stress ${{\varvec{\upsigma}}}$ as

2 $$\upmu \left( {{\mathbf{x}},{\text{t}}} \right) = \mathop \smallint \limits_{{0^{ - } }}^{{\text{t}}} {\mathbf{G}}\left( {{\text{t}} -\uptau } \right):\frac{{\partial {{\varvec{\upsigma}}}\left( {{\mathbf{x}},\uptau } \right)}}{{\partial\uptau }}{\text{d}}\uptau ,$$

being ${\mathbf{G}}\left( {{\text{t}} - {\uptau }} \right)$ a 4th order tensor, that we approximate as

3 $${\text{G}}_{{{\text{ijkl}}}} = {\upkappa }_{1} {\text{e}}^{{ - \frac{{{\text{t}} - {\uptau }}}{{{\uptau }_{1} }}{ }}} {\updelta }_{{{\text{ij}}}} {\updelta }_{{{\text{kl}}}} + \frac{1}{2}\left( {{\upgamma }_{0} + {\upgamma }_{1} {\text{e}}^{{ - \frac{{{\text{t}} - {\uptau }}}{{{\upnu }_{1} }}{ }}} } \right)\left( {{\updelta }_{{{\text{ik}}}} {\updelta }_{{{\text{jl}}}} + {\updelta }_{{{\text{il}}}} {\updelta }_{{{\text{jk}}}} } \right).$$

The parameters defining tensor ${\mathbf{G}}\left( {{\text{t}} - {\uptau }} \right)$ were considered to behave according to a Gaussian probability distribution, and unless indicated, they were modeled as uncorrelated. Estimated values were obtained from different animal models^20^, and are synthesized in Table 2.

The ossification process is assessed by defining a maturity distribution $\mathrm{M}\left(\mathbf{x},\mathrm{t}\right)$. This function can be interpreted as a scalar describing the physiological stage of a cell located at a point ${\mathbf{x}}$, in between the moment when it is born and the moment when it ossifies. It changes as:

4 $${\dot{\text{M}}}\left( {{\mathbf{x}},{\text{t}}} \right) = {\text{tr}}\left( {{\dot{\mathbf{\varepsilon }}}} \right)$$

The initial maturity distribution was defined as linearly varying with distance, being $\mathrm{M }= 0$ for the epiphyseal plate nodes in contact with the bones epiphyses and $\mathrm{M }= 1$ for the nodes in contact with the bone diaphysis^36^.

The numerical algorithm used for solving the model equations has been described in a previous publication^21^. This novel model requires performing an additional temporal integration for obtaining distortion term of Eq. (2), which required to introduce a slight software modification. More details, as well as a couple testing cases can be found in Sup. Mat. 5.

Boundary conditions for growth estimation were the same as for the determination of stresses in the epiphyseal plate. Bony regions were considered as rigid solids, and a Gaussian probability distribution was assumed for the epiphyseal growth plate Young Modulus ${\mathrm{E}}_{\mathrm{f}}$^3^. To model the normal use of a night-time orthotic, loading was considered to happen each day for a period ${\uplambda }_{\mathrm{use}}$ representing the time during which the patient sleeps, Table 2. This time was assumed to be normally distributed, in accordance with the current consensus about normal sleeping times for a 10 year old person^37^. Rotations along the y direction in sections a and b were used for quantification of changes in the anatomical lateral distal femoral (aLDFA) and lateral proximal tibial angles (aLPTA), Fig. 1. The summation of these two rotations was used as a measurement of the femorotibial angle achieved correction $\uptheta (\mathrm{t})$. For evaluating the effect of a hypothetical shape memory alloy based orthotic, the magnitude of the applied load was defined as a constant moment $\mathrm{T}(\mathrm{t}) = {\mathrm{T}}_{\mathrm{max}}$. These effects were compared against the produced by an orthosis in which applied load is supposed to be originated by the deformation of a linear elastic material, thus decreasing proportionally to the achieved correction $\theta (t)$ until reaching a desired correction ${\uptheta }_{0}$. In this case, applied effort was considered to vary as $\mathrm{T}(\mathrm{t}) = {\mathrm{T}}_{\mathrm{max}}[\frac{{\uptheta }_{0}-\uptheta \left(\mathrm{t}\right)}{{\uptheta }_{0}}]$. Growth dependence with desired correction was evaluated by assuming a Gaussian distribution for ${\uptheta }_{0}$, with a mean value similar to the typically treated with orthotic devices^1,4,9^, Table 2.

Two hundred runs were made in order to stochastically quantify growth in the knee. For each run, a particular set of model parameters was generated in accordance with Table 2. Growth was modeled for a 60 days period using the previously described^21^ explicit time integration method. Time step used in the growth algorithm was set at ∆t = 0.01 day, after a temporal convergence analysis (Sup. Fig. 2). The later was made for the mean value of the model parameters, and error for the computed angle was estimated to be smaller than 1%. Results can be reproduced with scripts provided in Sup. Mat. 6. For this case, the same converged mesh used for the determination of epiphyseal plates stresses was used.

The mean achieved change in femorotibial angle was fitted with the expression $\uptheta (\mathrm{t}) = {\mathrm{c}}_{1}\mathrm{t }+{\mathrm{c}}_{2}[\mathrm{exp}({\mathrm{c}}_{3}\mathrm{t})- 1]$ for the cases in which the applied load was constant, and $\uptheta (\mathrm{t}) = {\mathrm{c}}_{0}\{1-\mathrm{exp}\left\{{\mathrm{c}}_{1} +{\mathrm{c}}_{2}\left[\mathrm{exp}\left({\mathrm{c}}_{3}\mathrm{t}\right)-1\right]\right\}\}$ for the cases in which the applied load was considered as a function of achieved correction. Values of coefficients c_i_ were obtained by the least squares regression method. This allowed to extrapolate results for longer times than 60 days. Extrapolated results were used to compute the time in which a 50% of desired correction ${\uptheta }_{0}$ is achieved. Dependence of this characteristic time with the bones baseline growth speed, the sought correction ${\uptheta }_{0}$ and the time the patient wears the orthosis was analyzed. These results were used to estimate the benefits that could theoretically be achieved by designing a constant load orthosis, and how they depend on the desired final correction.

## Results

### Stress in the epiphyseal plate

**Figure 2:**
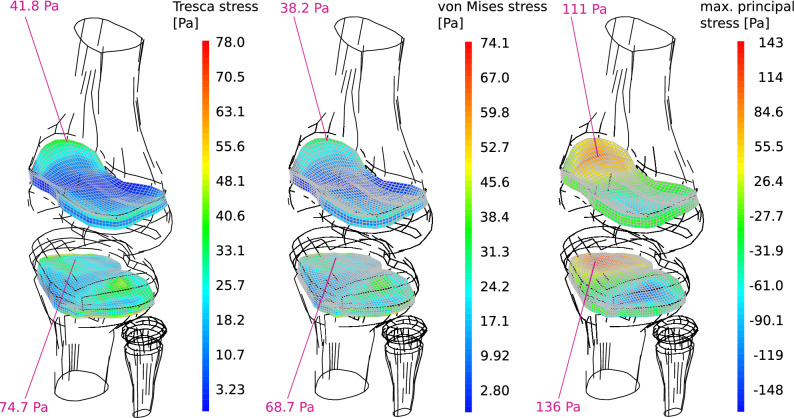
Stresses on the epiphyseal plate, for a unitary (1 Nmm) applied moment. Values and arrows in pink indicate the estimated stress peak level and its location, respectively. Maximum normal stress is the more conservative criterion for preventing epiphyseal damage, allowing a maximum applied moment of 1*.*1 Nm in order to not surpass a stress level of 0.153 MPa, considered to be safe^19^. Global error in the numerical solution was estimated in the order of 2% (Sup. Fig. 3), while material behavior related uncertainty in the computed stresses was estimated to be in the order of 1*.*5% (Sup. Fig. 4).

When a set of loads exerting a flexor moment for correcting genu varum is applied on the knee, stress distribution in the epiphyseal plates characterizes by the appearance of tensile stresses on the medial side of the physes, Fig. 2. Simultaneously, compressive stresses are generated in the lateral sides. Maximum stress is larger in the tibia physis, which can be explained by its reduced size as compared with the femur physis. Peak von Mises, as well as Tresca equivalent stresses are located near the perichondral ring. In the case of principal stresses, the peak values of this estimator are found at the interior of the epiphyseal plates, Fig. 2. The more conservative computed criterion for limiting the applied external flexor moment is to limit the maximum principal stress in order to not surpass the value of 0.153 MPa considered to be safe for epiphyseal distraction treatments^19^. This criterion yields a maximum applicable flexor moment of *T*_*max*_ = 1*.*1 Nm.

### Loads on the articular tissues

When considering the effects of peak physiological loads aligned with the leg mechanical axis, ligaments efforts are negligible. Stress intensity estimators such as von Mises stress, contact pressure and menisci deflection are larger towards the medial side of the knee. Estimated maximum solicitations for each relevant model region can be found in Sup. Table 1.

Mechanical solicitations computed for different load levels originated by an external orthotic can be found in Sup. Table 1. For these cases, negligible efforts arise in the medial side articular tissues. Contact forces arising on the lateral side of the knee are mainly balanced by a tensile load on the medial collateral ligament. This segment sustains a force approximately proportional to the applied flexor moment T, given by (6.4 m^−1^)T.

**Figure 3:**
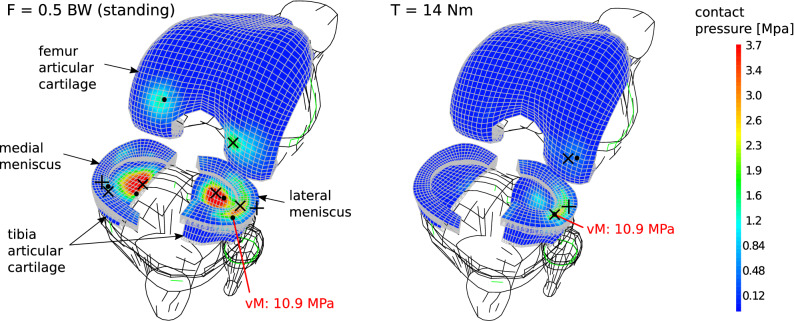
Comparison between the knee tissue stress estimators and deformations, for a typical load F occurring during still standing and for an “equally stressing” correcting moment T. •: location of maximum von Mises equivalent stress. × : location of maximum contact pressure. + : location of maximum menisci displacement. Tension in the medial collateral ligament due to the orthotic use was estimated in 77 N for this case.

Equivalent flexor moments, corresponding with different physiological loads can be defined based on a comparison of von Mises stress, contact pressure and deformation of relevant segments forming the knee joint. From these, it follows that a flexor moment of (14 ± 2) Nm stresses the knee as much as standing or cycling, in terms of the maximum von Mises stress on the lateral meniscus. A flexor moment of (18 ± 2) Nm provokes a maximum deflection and a maximum von Mises stress in the lateral meniscus equivalent to those occurring while walking, and a flexor moment of (22 ± 3) Nm causes similar deflections on this segment as jogging. The lower flexor moment inducing tissue mechanical solicitations equivalent to the ones that occur during a typical activity as still standing, Fig. 3, is an order of magnitude larger than the flexor moment of 1.1 Nm that was estimated to risk local damage in the epiphyseal plate.

Stresses in the articular cartilages and the menisci were obtained with a global estimated accuracy of 5%, which was achieved by using second order hexahedral elements (Sup. Mat. 8). An additional estimated error of 10% should be conferred due to variability in the geometrical model obtention^38^.

### Correction estimates

**Figure 4:**
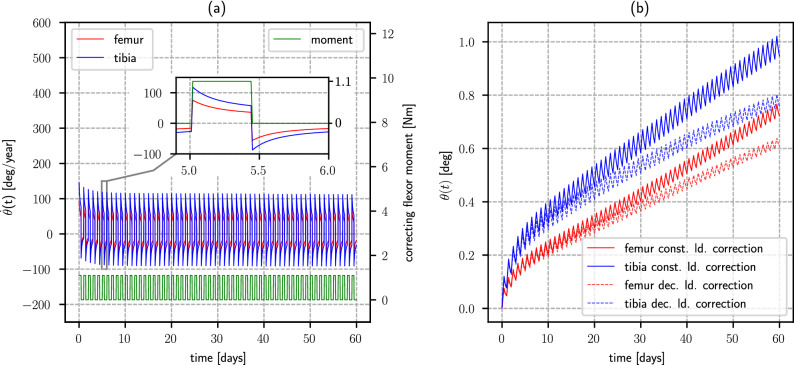
Typical correction evolution under a constant orthotic load. (**a**) Dependence of correction speed for the femur and the tibia, as a function of time and applied flexor moment. (**b**) Evolution of the femur and the tibia correction angle. Notice how the model predicts an initial, more rapid mean correction rate, that stabilizes at around 10 days of treatment in the case of a constant load orthotic, progressively decreasing in the case of a decreasing load orthotic with a goal correction ${\theta }_{0}$ = 2 deg.

The computed effect of using a constant load orthosis at night on the knee bones angular correction is shown in Fig. 4. The plotting corresponds with the case for which all the model parameters adopt their mean value. Model-predicted correction speed characterizes for showing transient features that can be analyzed in terms of two characteristic time scales, Fig. 4a. At the moment the load is applied or removed, it instantaneously changes to later approach an asymptotic value in an exponential-like fashion, as shown in the detail of Fig. 4a. In a weeks long time scale, the mean correction speed shows a decreasing behavior, until reaching a stable value. Predicted change in angular deviation typically behaves as shown in Fig. 4b. This comportment can be conceived as an initial period of about 10 days, in which the mean correction speed decreases until reaching a steady value. The mean angular correction can be adequately described (*r* ≥ 0*.*99 for the whole set of analyzed cases) with an expression of the type $\theta (t) = {c}_{1}t +{c}_{2}[\exp({c}_{3}t)- 1]$. The instantaneous computed correction shows a swaying behavior around this mean value, that can be directly linked to the transient effects arising each day when fitting or removing the orthosis. These day-long transient effects represent approximately 5% of the mean correction in a 60 days period.

**Figure 5:**
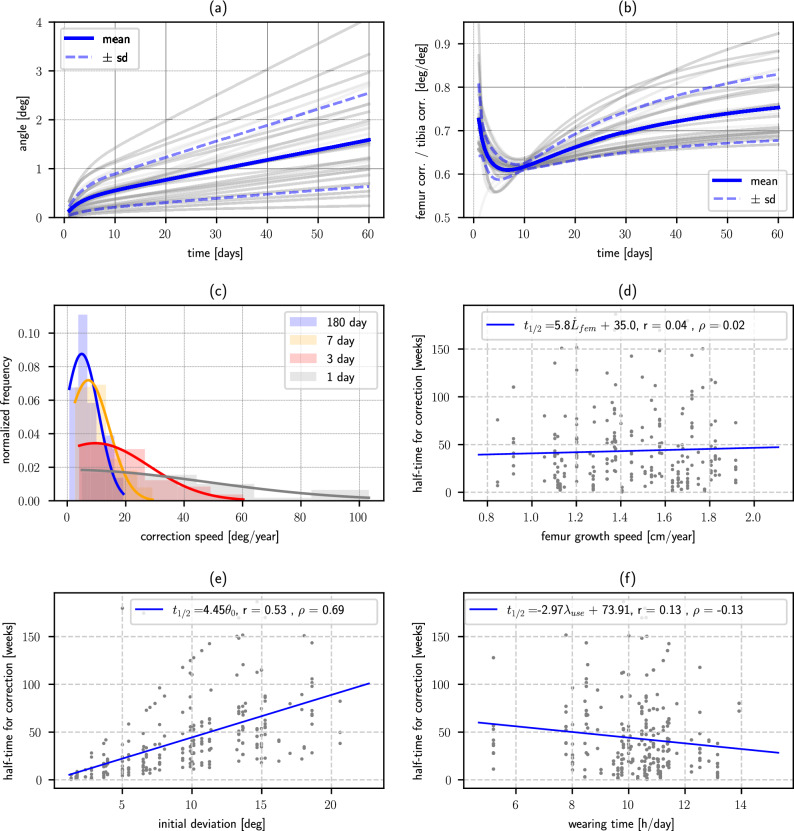
Growth evolution quantifiers in a set of 200 stochastic runs, for an orthotic applying a constant correcting load. (**a**) Change in the knee deviation angle evolution. (**b**) Femur versus tibia correction ratio evolution. (**c**) Correcting speed pdf. as a function of time. (**d**) Dependence of correction time with growth speed. (**e**) Dependence of correction time with initial deviation. (**f**) Dependence of time for correction with the time the patient uses the orthotic.

The mean change in femorotibial angle was computed for a set of 200 stochastic runs, with a constant applied load, Fig. 5a. The mean achieved correction is indicated, as well as the corresponding standard deviation. Although the values of the achieved correction vary, the behavior of the corresponding responses does not substantially differ from the previously described. Correction in the femur is in all cases smaller than in the tibia, Fig. 5b, asymptotically converging to represent a (76 ± 10)% of the achieved in the last. Femorotibial angle correction speed decreases with time, converging towards a stable value, Fig. 5c, as well as its deviation from the computed mean value. The asymptotic probability distribution of this quantity has a mean value of 7*.*4 deg/year with a standard deviation of 4*.*6 deg/year. The characteristic correction time was found to be uncorrelated (*r* = 0*.*04*, ρ* = 0*.*02) with the bones longitudinal growth speed for the considered range, Fig. 5d. A strong, positive correlation (*r* = 0*.*53*, ρ* = 0*.*69) was found between this time and the desired correction *θ*_0_, Fig. 5e. A slight, although insignificant (*r* = 0*.*13*, ρ* = − 0*.*13), negative correlation was found with wearing time, Fig. 5f.

When the applied load decreases in proportion to the achieved correction, the model predicted behavior shows an initial, more rapid correction speed, as in the previous case. During the first few days, correction is very similar to the one achieved with a constant load orthotic, although it slows down as time advances, due to the decay in the applied load, Fig. 4b. In all cases, this causes the correction speed to progressively decrease as time advances, Sup. Fig. 5a. Correction of the femur is smaller than correction of the tibia, Sup. Fig. 5b, asymptotically converging to represent a (78 ± 10)% of the angular correction achieved in the last. Angular correction speed is not very different from the obtained with a constant-load orthosis during the first week of treatment, although it continuously decreases with time, Sup. Fig. 5c. Probability distribution of this quantity has a mean value of 3*.*9 deg/year with a standard deviation of 2*.*2 deg/year after 180 days of treatment, and tends towards $\delta (0)$ as time increases. As with constant-load orthosis, the characteristic correction time had no significant (*r* = 0*.*04*, ρ* = 0*.*03) correlation with the bones longitudinal growth speed, Sup. Fig. 5d. A strong positive correlation (*r* = 0*.*53*, ρ* = 0*.*69) was found between the time to achieve half the goal correction *θ*_0_ and its value, Sup. Fig. 5e, and an insignificant (*r* = 0*.*13*, ρ* = − 0*.*13) dependence of this parameter with wearing time is observed, Sup. Fig. 5f.

**Figure 6:**
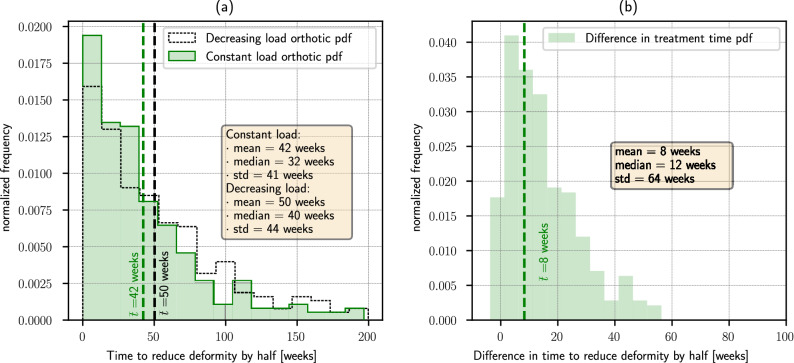
Time necessary to reduce an initial knee deviation by half. (**a**) Comparison of the pdf of necessary time, for a constant load and a decreasing load orthotic. (**b**) Pdf of the difference in wearing time for a constant load and a decreasing load orthotic. With a probability greater than 50%, treatment time for reducing deformity by half is 12 weeks shorter with a constant load orthotic.

Figure 6a shows the computed probability density function of the time necessary to achieve half of the goal correction ${\theta }_{0}$. With a constant load orthotic, the mean characteristic time is in the order of 42 weeks, while for a decreasing load orthotic this time is increased to 50 weeks. Figure 6b shows the pdf of the difference in time necessary to reduce a given deviation ${\theta }_{0}$ by half, for a constant load and a decreasing load orthosis. The mean value of this reduction is in the order of 8 weeks. In half of the computed cases, this characteristic time is at least 12 weeks shorter with a constant load orthosis than with a decreasing load orthosis.

The fraction of time required to achieve a given correction ${\theta }_{0}$ with a decreasing load orthotic, as represented by the time required with a constant load orthotic, gives information about the improvements that could be obtained by assuring a constant load is applied throughout the orthotic treatment. This fraction depends on the stage at which it is evaluated, and has a positive correlation (*ρ* = 0*.*6) with the goal correction ${\theta }_{0}$, although it does not strongly vary in the studied range, Sup. Fig. 6. Roughly, the treatment time required to produce a correction representing 20, 50 and 80% of the goal angle ${\theta }_{0}$ with a constant load orthosis is at most the 90%, 72% and 50% of the time estimated for a decreasing load orthosis, respectively. This quantity decreases as the treatment progresses, i. e., when approaching the desired correction, the required treatment time with a constant load orthosis represents a smaller fraction of the time that would be necessary to achieve the same correction with a decreasing load orthosis.

## Discussion

Shape memory alloys have pseudoelastic properties that can potentially be used to create orthopedic devices able to exert prescribed, rather unchanging loads. Its use could hypothetically be a significant improvement for current orthotic treatments, somehow comparable with the introduction of NiTi arcs in orthodontics^13,39^. The precise quantification of the forces to be applied is a key point for this^18^. These should be as high as possible to accelerate correction, but at the same time must not injury the patient. Here, we focus on the lateral deviations of the knee, because to date these are the only kind of pathologies that have been successfully treated with external ortheses^1,9^. Treatment of other common deviations such as tibial anteversion, femoral anteversion or recurvatum would arguably be impossible without directly intervening onto the affected bone because of the physical limitations existing for effectively transferring the necessary mechanical loads to the treated epiphyseal plates. Sup. Fig. 7a illustrates the case for when a bone anteversion is to be corrected, in which applied torque would likely induce a joint dislocation without effectively transferring the intended load to the epiphyseal plates. Sup. Fig. 7b illustrates the case for which a recurvatum deformity is to be treated. In such case, the knee would be flexed without exerting a correcting effort.

To date, orthotic management of knee lateral deviations remains controverted among existing growth modulation methods^6,7^. A critical feature of these is that they are only applicable during a time window comprising childhood and early adolescence, in which it is necessary to make the most of the available time^4^. For this reason, when there is a risk of orthotic treatments being insufficient to achieve a desired correction before epiphyseal closure, minimally invasive surgeries are preferred, as Blount staples^2,40^ or eight-plates implantation^41,42^. When these alternatives fail, the conventionally accepted technique for correcting bone deformities consists in performing an osteotomy, which is considered to be a highly invasive procedure^1,4,5^. This makes an adequate treatment planning to be of upmost importance, hence the relevance of the present study.

Bone growth is often studied on simplified geometrical models^3,43–45^. This can markedly affect quantitative mechanobiological predictions^43^. In the present work, we studied the patient specific knee geometry of a 10 years old female. Despite the fact that the patient did not have a pathological tibiofemoral angle deviation, the effects of applied loads were hypothesized to be independent of the initial deformity, provided that the studied pathology characterizes by an idiopatic lateral deviation of the knee, anatomical structures are preserved and growth is otherwise normal. Obtained results are thus invalid for cases in which the knee deviation has another root cause, e.g., the existence of an epiphyseal bridge^1^.

Although this real-like geometry arguably allows to reduce associated errors as much as possible with the current state of the art, limitations on the following analysis subsist due to geometrical differences arising for different patients. Study of effects due to this source of uncertainty remains a matter of future research. Results suggest that although following qualitative observations could arguably be extended to other patients, a precise determination of applicable load values should be performed for each individual in consideration to their particular anatomical features.

In order to reduce undesired effects, orthoses should be designed to minimize mechanical efforts beyond those aiming to the sought therapeutic effect^9^. On these terms, external loads provoking ligament efforts for laterally stabilizing the knee would not be desirable. Also, being mainly orthogonal to the physes section, its effects on the sought femorotibial angle correction would arguably be negligible. External forces are balanced by a contact force on the lateral side of the knee and a tensile effort on the medial ligament, as we verified here via finite elements computations. This simplification allows to model efforts produced by knee orthosis as two same magnitude, counteracting moments exerted on the femur and the tibia.

For this particular patient, applied flexor moment should not exceed 1*.*1 Nm in order to avoid stress injuries on the epiphyseal plate. Such injuries are considered to occur when the forces applied over time exceed the ability of the tissue to repair itself^1^ and should not be mistaken with more acute lesions as epiphyseal fractures^46^. Scarce experimental information exists for humans on this topic, and there are not well established technical bioengineering criteria for determining the onset of stress injuries^1,19,47^. Existing data indicates that the physis can sustain distractive loads up to between 0*.*153 and 0*.*296 N/mm^2^ before epiphysiolisis^19^. These values are similar to the stress of 0*.*2 MPa typically applied to study growth in animal models^48–51^.

We evaluated three commonly accepted material failure criteria in order to bound the applicable orthotic efforts, finding the limitation of the maximum principal stress to be the more conservative criterion. Flexor moment obtained on this manner might be largely underestimated, as supported by several observations. Firstly, the stress values reported in the literature were obtained as an average between the force applied on the epiphyseal plate and its transverse area, thus local stresses could be significantly higher when epiphysiolisis occurs. The maximum equivalent stress was conservatively bounded to 0*.*153 MPa, although it could be safely increased up to 0*.*296 MPa for some patients^19^. Also, the possibility of maximum principal stress criterion being over-conservative should be pondered, because moment could be increased by a factor of roughly 2 if the von Mises or the Tresca stresses are found to be better estimators of the stress injury condition. In consequence, a constant moment up to 3*.*7 Nm could be safely applied on this patient. At last, in the present work we have neglected the loads sustained by other tissues adjacent to the physis, as the perichondral ring, the skin, the synovial capsule, etc. For this reason, the load effectively transmitted to the epiphyseal plates would presumably be a fraction of the externally applied load.

A relevant result of the present study relates with mechanical solicitations on the articular tissues. It is important to notice that, as there are not well established technical bioengineering criteria for their determination, especially during childhood^1,32^, this task required to compute several stress intensity estimators occurring during normal activities. The selection of a normal patient for the study was made in consideration of this. The computation of applicable stress levels after data obtained for a pathological patient would not be safe, because lateral deviations of the knee have been proven to increase the stress on the articular tissues, and this is known to induce long-term damage such as arthrosis^52^. An applied moment of roughly 10 times the magnitude estimated to be at risk of producing stress injuries on the physeal cartilage causes solicitations similar to those occurring while the person performs a typical activity as standing. At the same time, equivalent stresses predicting material failure of the physeal cartilage are in the order of 3 MPa^32,53^, which also represents about 10 times the maximum stress that has been found to produce epiphysiolisis under sustained loading^19^. Maximum von Mises stress on the menisci was estimated in 12 MPa, for a mechanical load representing the peak force occurring while jogging. This stress is roughly 3 times smaller than the yield stress of the menisci tissue^31,53^, and one order of magnitude larger than the stress caused by an applied moment of 1*.*1 Nm. These results suggest that although the knee is able to tolerate substantial efforts for short periods of time, loads about an order of magnitude smaller than the ones appearing during typical activities are prone to overcome the tissues self-repairing capacity, when continuously applied for long periods. Daily use of a device exerting these constant loads would presumably not induce any relevant fatigue effect on the affected tissues, given that this would arguably require only one loading cycle per day. Such conclusion is strengthened by observing that computed loading levels are about an order of magnitude smaller than the ones typically sustained by the knee during normal activities that cause alternating loading.

The lack of human growth mechanobiological data constitutes an important limitation of the present study. Growth model parameters values were obtained from a large collection of published experiments on animal models^20^. Results are sustained by existing evidence indicating that mechanobiological effects of applied stress are not significantly different for the same bones of rats, rabbits and calves, suggesting the possibility of this dependence being the same for humans^48^.

For a constant load orthosis, after an initial adaptation period of approximately 10 days, the model-predicted mean correction rate stabilizes at 0.35 deg/month for the tibia and 0.26 deg/month for the femur. These correction rates are similar to the achieved with other guided growth treatments based on devices that are inserted directly onto the affected bones^3,41,42^. For a decreasing load orthotic, correction rates continue to decrease as time advances. After three months of treatment, the mean correction rate reaches 0.18 deg/month for the tibia, and 0.14 deg/month for the femur. This suggests that it could be possible to avoid the necessity of surgically treating some knee deformities if orthosis effectiveness is to be increased by assuring the application of a constant, prescribed load. The angular evolution observed in Fig. 4b, in which a more rapid correction speed is shown at the beginning of the treatment stabilizing afterwards, raises the question whether or not suspending orthoses use at regular intervals and/or varying the loading levels in a certain prescribed manner could significantly improve the achieved correction rate. Also, the increase in width of the epiphyseal plate within the studied time lapse is negligible as compared with the total increase in length of the studied bone segments. Nevertheless, the bone increases in size as time proceeds^54^. For this reason, applied loads should arguably be progressively increased when treatment time is considerably long, so as maximum principal stress on the physis is not decreased because of these geometrical changes. We believe these observations suggest a direction for future investigations.

Previous studies have shown that mechanically induced growth changes are proportional to the loading time^55^. Here, we found that, within normal sleeping times, changes in achieved correction because of this variable are not significant. It should be noticed that this does not necessarily imply that correction cannot be increased if the orthotic is worn during longer periods than studied here. Similarly, the achieved angular correction does not vary significantly (r = 0.04) with bone growth speed, when considering this variable to fall within its normal values for a 10 years old person.

The tibia physis in our model is smaller in size than the femur physis. This explains why the maximum stress occurs on the first, as well as why the estimated angular correction is about 25% larger in the tibia. This result suggests that orthotic prescription should be reserved for those cases in which femorotibial angle deformities are originated both in the anatomical lateral deflection femur angle (aLDFA) and the anatomical lateral deflection tibia angle (aLPTA), and specially if desired correction in aLPTA is larger than in aLDFA.

The improvement in treatment time for reducing a given deviation by half was estimated to have a median of 12 weeks with a constant load orthotic, while the mean time for achieving the same correction with a conventional orthotic was estimated to be of 50 weeks. This represents a relative reduction in time in the order of 25%. This reduction is strongly correlated with the desired correction, although the benefits of using a constant load orthotic are predicted to be only slightly larger in the treatment of smaller deviations.

## Conclusion

Two relevant aspects are to be assessed before any new orthotic device can be conceived and later worn by a patient: applicable load levels should be quantified and limited to avoid damage in the involved bones and joints; also treatment effectiveness should be estimated, in order to ponder possible alternatives in the patients best interest. In the present study, which has been particularized for a 10 year old female, patient-specific knee model, we found that:

- Based on a maximum principal stress criterion on the epiphyseal plates, orthosis for correcting knee lateral deviations should exert a flexor moment on the knee not greater than 1*.*1 Nm in order to avoid the possibility of stress injury in the epiphyseal plates. This moment could be increased up to 3*.*7 Nm if less conservative criteria are adopted.
- For short periods of time, the knee could be able to sustain up to about 10 times these loads, without exceeding the tissue self-repairing capacity.
- Application of a constant flexor moment of 1*.*1 Nm could change femorotibial angle at a constant rate of (7*.*4 ± 4*.*6) deg/year, after a transient period in the order of a couple weeks. This is similar to correction rates achieved by more invasive growth modulation treatments.
- A load decreasing in proportion to achieved correction does not reach a constant correction rate. After 180 days, it is reduced to (3*.*9 ± 2*.*2) deg/year, asymptotically decreasing to 0. This is roughly half of the correction rates achieved by other growth modulation treatments.
- Lateral distal femoral angle correction represents in the order of (75 ± 10)% of the correction of the lateral proximal tibial angle, independently of the characteristics of applied load.
- For femorotibial deviations of (10 ± 5) deg (mean ± std), treatment time for reducing the deformity by half was estimated to be, with a probability greater than 50%, 12 weeks shorter with a constant load orthotic than with a decreasing load orthotic. This represents a 24% improvement when considering that the mean required time for this was estimated at 50 weeks for a decreasing load orthotic.

## Supplementary Information

Supplementary Information.

## Data Availability

All data used in this study is provided as supplementary material.
