# Supplementary material for: A numerical study towards shape memory alloys application in orthotic management of pediatric knee lateral deviations
Source: Sci Rep. 2023 Feb 6;13:2134. doi: 10.1038/s41598-023-29254-z (PMC9902535; doi:10.1038/s41598-023-29254-z)
Supplement: Supplementary file 1 — Supplementary Information. [file 41598_2023_29254_MOESM1_ESM.zip › Sup_mats/LIST_OF_MATERIALS.pdf]

List of supplementary materials

- **Sup. Mat. 1:** Converged mesh used for stress and growth computations.
- **Sup. Mat. 2:** Converged mesh used for articular tissues stresses computations.
- **Sup. Mat. 3:** Scripts used for computing epiphyseal plates stresses.
- **Sup. Mat. 4:** Scripts used for computing articular tissues stresses.
- **Sup. Mat. 5:** Numerical method for integration of growth equations and code verification cases.
- **Sup. Mat. 6:** Script for computing induced growth.
- **Sup. Mat. 7:** Updated ad-hoc software for growth and remodeling computing.
- **Sup. Mat. 8:** Solution convergence and error estimation of stress in the articular tissues.

- **Sup. Fig. 1:** Free body analysis of the knee joint.
- **Sup. Fig. 2:** Growth temporal convergence analysis.
- **Sup. Fig. 3:** Solution convergence of stress in the epiphyseal plate.
- **Sup. Fig. 4:** Uncertainty quantification in the maximum applicable flexor moment based on epiphyseal stresses.
- **Sup. Fig. 5:** Growth evolution quantifiers for a decreasing load orthotic.
- **Sup. Fig. 6:** Fraction of treatment times required with a constant load orthosis and a decreasing load orthosis.
- **Sup. Fig. 7:** Effect of applied loads intended for (a) correcting a femoral/tibial anteversion (b) correcting a recurvatum bone deviation.

- **Sup. Tab. 1:** Computed mechanical solicitations on the knee, for physiological and orthotic loads.
