# Supplementary material for: A numerical study towards shape memory alloys application in orthotic management of pediatric knee lateral deviations
Source: Sci Rep. 2023 Feb 6;13:2134. doi: 10.1038/s41598-023-29254-z (PMC9902535; doi:10.1038/s41598-023-29254-z)
Supplement: Supplementary file 1 — Supplementary Information. [file 41598_2023_29254_MOESM1_ESM.zip › Sup_mats/Sup_Fig_1.pdf]

# Free body analysis and simplifications made for defining model boundary conditions.

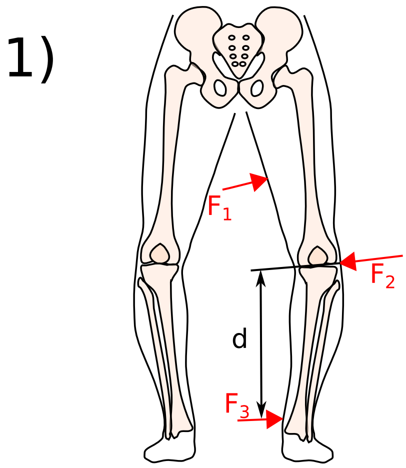


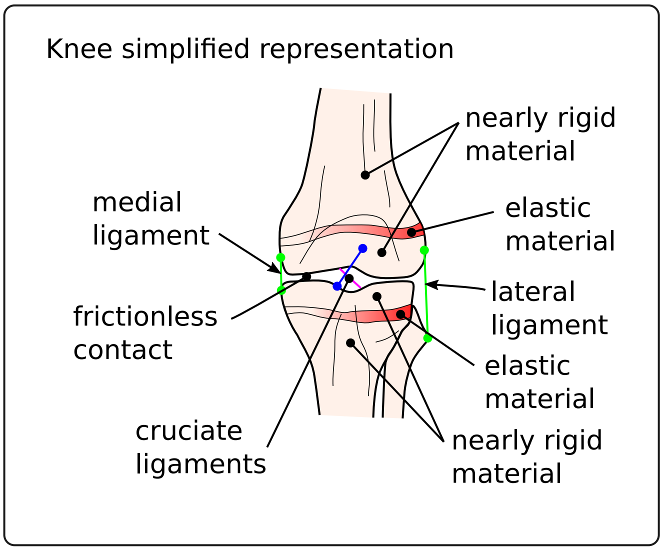


Orthosis effect is equivalent to a set of three forces applied on the leg. A flexor moment $T = F_3.d$ is sustained at the knee level.

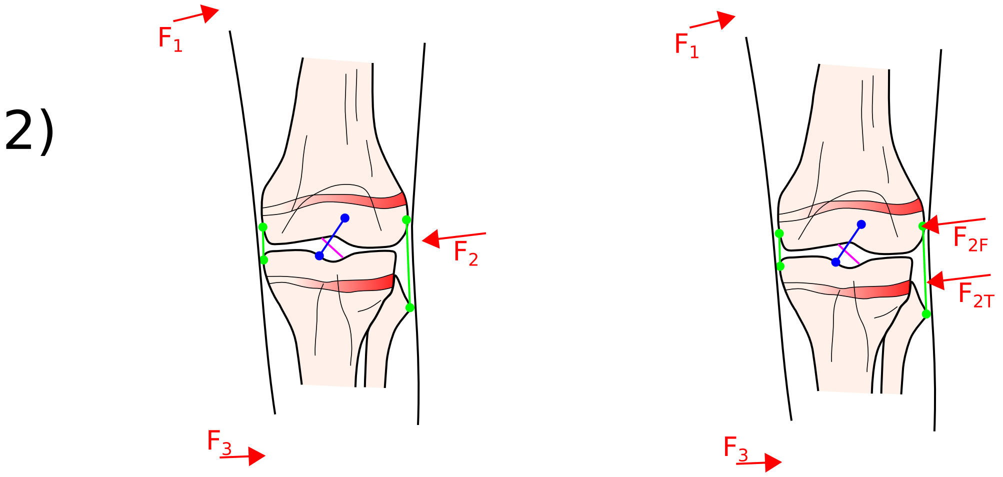


The orthotic is required to be designed in such a manner that lateral efforts, tending to produce a joint dislocation are negligible. Also forces tending to distract the joint are supposed to be minimized. Under these hypothesis, force $F_2$ is sustained both between the femur and the tibia, giving rise to forces $F_{2F}$ and $F_{2T}$.

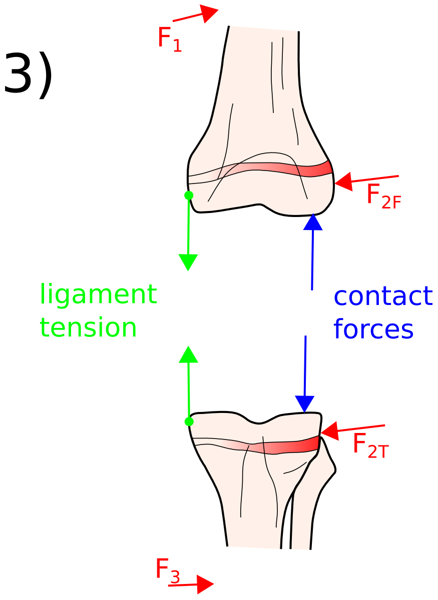


External efforts are balanced by the medial ligament tension and the contact forces on the articular cartilage.

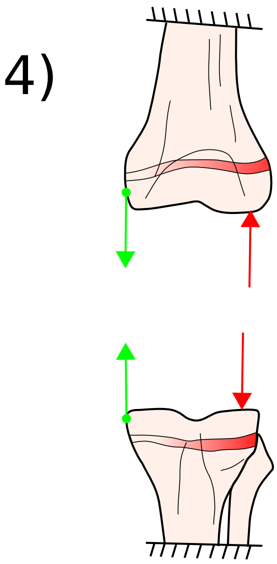


Forces equilibrium can be obtained by imposing a no displacement condition as indicated, and considering the action of contact forces and ligament tension.

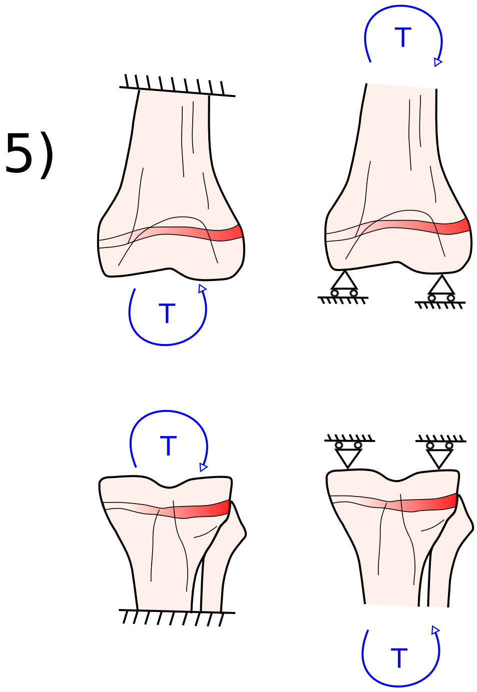


The effect of external forces can thus be reduced to a couple of moments T equal and opposite acting on the bones diaphyses.
