# Supplementary material for: A numerical study towards shape memory alloys application in orthotic management of pediatric knee lateral deviations
Source: Sci Rep. 2023 Feb 6;13:2134. doi: 10.1038/s41598-023-29254-z (PMC9902535; doi:10.1038/s41598-023-29254-z)
Supplement: Supplementary file 1 — Supplementary Information. [file 41598_2023_29254_MOESM1_ESM.zip › Sup_mats/Sup_Fig_3.pdf]

Mesh subdivisions performed for convergence analysis.

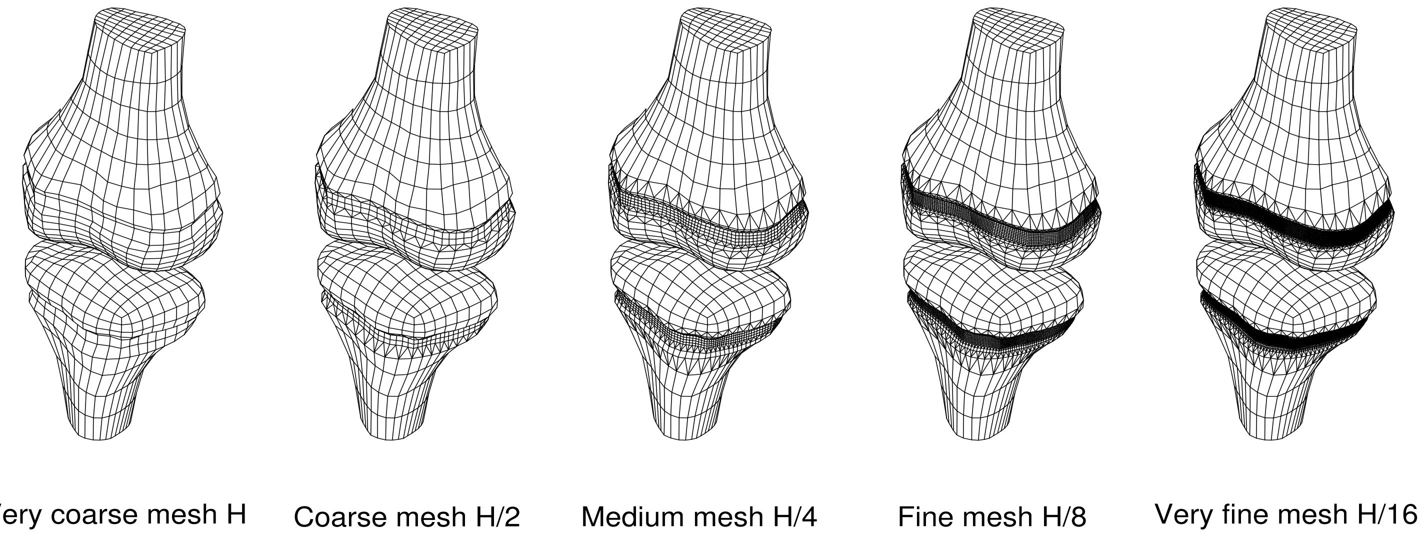

Global percent error in the stress of obtained solution.

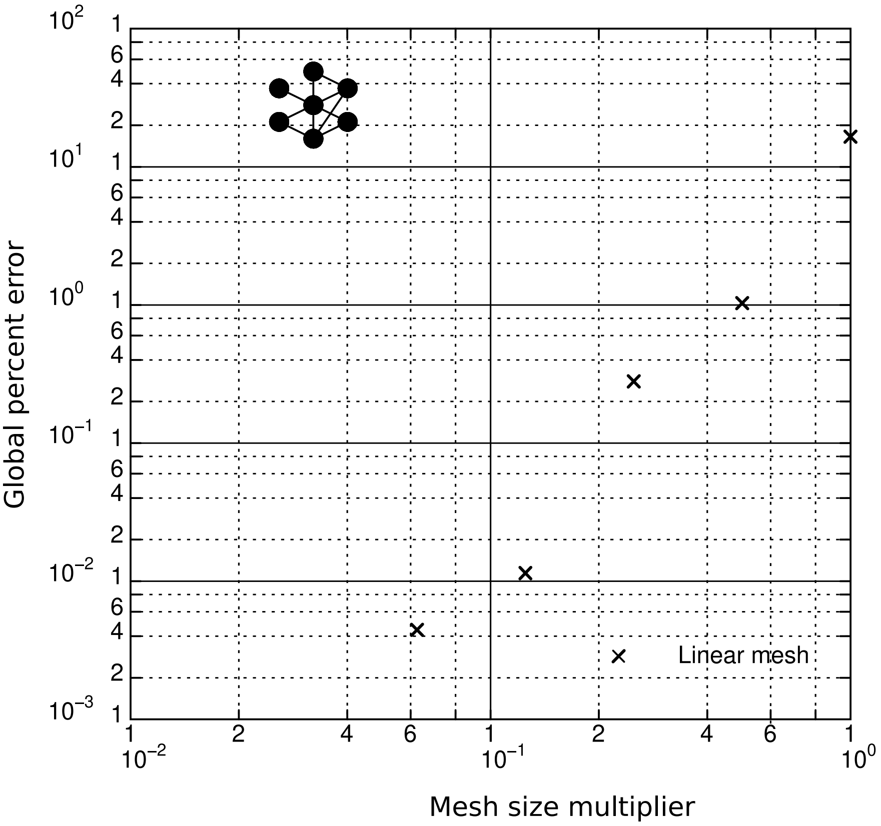

Global error was computed as:

$$\varepsilon_{\%} = max(\sqrt{\int_{\Omega} \frac{(\sigma_{ij}-\sigma_{ij}^{0})}{\sigma_{ij}^{0}} dV}) \times 100\%$$

having estimated σ_{ij}^{0} from the Richardson extrapolation of the three higher refinement meshes solution.
