# Supplementary material for: A numerical study towards shape memory alloys application in orthotic management of pediatric knee lateral deviations
Source: Sci Rep. 2023 Feb 6;13:2134. doi: 10.1038/s41598-023-29254-z (PMC9902535; doi:10.1038/s41598-023-29254-z)
Supplement: Supplementary file 1 — Supplementary Information. [file 41598_2023_29254_MOESM1_ESM.zip › Sup_mats/Sup_Mat_5.pdf]

Integration scheme and code test cases

August 25, 2022

Abstract

This document describes the numerical algorithm used for incorporating the growth model into software described in [2]. Although the same global algorithm is used, a few subtle modifications have been made in the manner in which growth velocities are computed. Here, particular details and code test cases are provided.

1 Numerical integration scheme

As described in a previous work [2], using a generic bone growth model requires to specify a growth speed tensor $\dot{\varepsilon}(\mathbf{x}, t)$, at a given time t and for every point of the considered domain $\boldsymbol{x}$. This growth speed is used to compute a set of equivalent nodal forces, which are the input to compute the displacements resulting from growth. Code provided in [2] was implemented and validated for a simple growth model, for which growth speed was obtained as a function of stress in the previous time-step, by using an explicit time integration scheme. In the present work, growth speed is computed as:

$$\dot{\boldsymbol{\varepsilon}}(\boldsymbol{x}, t) \quad = \quad \dot{\boldsymbol{\varepsilon}}^{\mathbf{0}}(\boldsymbol{x}, t) + \dot{\varepsilon}_B \int_{0^-}^{t} \boldsymbol{G}(t-\tau) : \dot{\boldsymbol{\sigma}}(\boldsymbol{x}, \tau) \mathrm{d}\tau, \tag{1}$$

being $\dot{\boldsymbol{\varepsilon}}_B$ a prescribed (constant) tensor, $\dot{\boldsymbol{\sigma}}(\boldsymbol{x}, \tau)$ the rate of change in applied stress at a given time τ and $\boldsymbol{G}(t-\tau)$ a 4th order tensor, shaped as:

$$\boldsymbol{G}(t-\tau) \quad = \quad \kappa_1 e^{-\frac{t-\tau}{\tau_1}} \delta_{ij}\delta_{kl} + \frac{1}{2}(\gamma_0 + \gamma_1 e^{-\frac{t-\tau}{\nu_1}})(\delta_{il}\delta_{kj} + \delta_{ik}\delta_{lj}). \tag{2}$$

This form of tensor $\dot{\boldsymbol{\varepsilon}}(t)$ is rather complex, and numerical integration of Eq. 1 cannot be done immediately in a computationally efficient manner, because it would require to compute and save all stresses for each time step previous to the computed stress t. This is due to the fact of the integrand of Eq. 1 being the parameter τ instead of the time t.

To overcome this difficulty, three fields μ_1, μ_2 and μ_3 were defined as:

$$\begin{aligned}
\mu_{kl}^{(1)} &= \int_0^t \kappa_1 e^{-\frac{t-\tau}{\tau_1}} \delta_{ij}\delta_{kl}\dot{\sigma}_{ij}(\tau)\mathrm{d}\tau \\
\mu_{kl}^{(2)} &= \int_0^t \frac{1}{2}\gamma_0(\delta_{il}\delta_{kj} + \delta_{ik}\delta_{lj})\dot{\sigma}_{ij}(\tau)\mathrm{d}\tau \\
\mu_{kl}^{(3)} &= \int_0^t \frac{1}{2}\gamma_1 e^{-\frac{t-\tau}{\nu_1}}(\delta_{il}\delta_{kj} + \delta_{ik}\delta_{lj})\dot{\sigma}_{ij}(\tau)\mathrm{d}\tau.
\end{aligned}$$

Differentiation of these fields allows to write:

$$
\begin{aligned}
\frac{\partial \mu_{kl}^{(1)}}{\partial t} &= \int_0^t -\frac{\kappa_1}{\tau_1} e^{-\frac{t-\tau}{\tau_1}} \delta_{ij}\delta_{kl}\dot{\sigma}_{ij}(\tau)\mathrm{d}\tau + \kappa_1 \delta_{ij}\delta_{kl}\dot{\sigma}_{ij}(t) - \kappa_1 e^{-\frac{t}{\tau_1}} \delta_{ij}\delta_{kl}\dot{\sigma}_{ij}(0) \\
\frac{\partial \mu_{kl}^{(2)}}{\partial t} &= \frac{1}{2}\gamma_0(\delta_{il}\delta_{kj} + \delta_{ik}\delta_{lj})\dot{\sigma}_{ij}(t) - \frac{1}{2}\gamma_0(\delta_{il}\delta_{kj} + \delta_{ik}\delta_{lj})\dot{\sigma}_{ij}(0) \\
\frac{\partial \mu_{kl}^{(3)}}{\partial t} &= \int_0^t -\frac{1}{2}\frac{\gamma_1}{\nu_1} e^{-\frac{t-\tau}{\nu_1}} \delta_{ij}\delta_{kl}\dot{\sigma}_{ij}(\tau)\mathrm{d}\tau + \frac{1}{2}\gamma_1(\delta_{il}\delta_{kj} + \delta_{ik}\delta_{lj})\dot{\sigma}_{ij}(t) \\
&- \frac{1}{2}\gamma_1 e^{-\frac{t-\tau}{\nu_1}}(\delta_{il}\delta_{kj} + \delta_{ik}\delta_{lj})\dot{\sigma}_{ij}(0). \qquad (3)
\end{aligned}
$$

By assuming that $\dot{\boldsymbol{\sigma}}(0) = \mathbf{0}$ (which can always be achieved by adequately defining the initial time for computation), Eqs. 3 can be written as:

$$
\begin{aligned}
\frac{\partial \mu_{kl}^{(1)}}{\partial t} &= -\frac{1}{\tau_1}\mu_{kl}^{(1)} + \kappa_1 \delta_{ij}\delta_{kl}\dot{\sigma}_{ij}(t) \\
\frac{\partial \mu_{kl}^{(2)}}{\partial t} &= \frac{1}{2}\gamma_0(\delta_{il}\delta_{kj} + \delta_{ik}\delta_{lj})\dot{\sigma}_{ij}(t) \\
\frac{\partial \mu_{kl}^{(3)}}{\partial t} &= -\frac{1}{\nu_1}\mu_{kl}^{(3)} + \frac{1}{2}\gamma_1(\delta_{il}\delta_{kj} + \delta_{ik}\delta_{lj})\dot{\sigma}_{ij}(t). \qquad (4)
\end{aligned}
$$

These equations can be explicitely integrated in the numerical scheme, by computing, for each time t_i, the quantities:

$$
\begin{aligned}
\mu_{kl}^{(1)}(t_i) &= \mu_{kl}^{(1)}(t_{i-1}) - \frac{1}{\tau_1}\mu_{kl}^{(1)}(t_i - t_{i-1}) + \kappa_1 \delta_{ij}\delta_{kl}[\sigma_{ij}(t_i) - \sigma_{ij}(t_{i-1})] \\
\mu_{kl}^{(2)}(t_i) &= \mu_{kl}^{(2)}(t_{i-1}) + \frac{1}{2}\gamma_0(\delta_{il}\delta_{kj} + \delta_{ik}\delta_{lj})[\sigma_{ij}(t_i) - \sigma_{ij}(t_{i-1})] \\
\mu_{kl}^{(3)}(t_i) &= \mu_{kl}^{(3)}(t_{i-1}) - \frac{1}{\nu_1}\mu_{kl}^{(2)}(t_i - t_{i-1}) + \frac{1}{2}\gamma_1 \delta_{ij}\delta_{kl}[\sigma_{ij}(t_i) - \sigma_{ij}(t_{i-1})].
\end{aligned}
$$

This requires to keep in the computer memory only the fields $\mu_{kl}^{(1)}(t_i), \mu_{kl}^{(2)}(t_i), \mu_{kl}^{(3)}(t_i)$ and $\sigma_{ij}(t_{i-1})$, which results in a more efficient computational solution. Then, growth speed can be computed as:

$$
\dot{\boldsymbol{\varepsilon}}(t_i) = \dot{\boldsymbol{\varepsilon}}^{\mathbf{0}}(\boldsymbol{x}, t_i) + \dot{\varepsilon}_B[\boldsymbol{\mu}^{(1)}(t_i) + \boldsymbol{\mu}^{(2)}(t_i) + \boldsymbol{\mu}^{(3)}(t_i)].
$$

After obtaining $\dot{\boldsymbol{\varepsilon}}(t_i)$, computing algorithm proceeds as described in [2].

2 Validation cases

Two validation cases were selected for testing the used software. The first corresponds with simplified geometries subjected to uniaxial loads, in approximately plain deformations stress states. These cases were selected as they allow to verify that the growth terms dependent on the principal normal stresses are correctly computed. For verifying that the growth terms dependent on the shear applied stresses are correctly computed, a cylindrical geometry subjected to a torsional load was studied, and results were compared with available analytical solutions for this case.

2.1 Uniaxial uniform loads

Consider an epiphyseal plate of thickness e, subjected to a uniaxial stress σ_1 applied instantaneously at $t = 0$ in the direction of longitudinal growth. Consider also the epiphyseal plate dimensions in directions 2 and 3 being significantly larger than e, the epiphyseal cartilage a linear elastic material of Young modulus E and Poisson ratio ν , and the bony tissue a nearly rigid material. Under these circumstances, growth in directions 2 and 3 is restrained. Shear stresses are nil, and so are growth speed tensor $\dot{\varepsilon}(t)$ extra-diagonal elements.

Stress tensor is such that:

$$\begin{aligned}\dot{\sigma}_{11}(t) &= \delta(0)\sigma_1\\ \dot{\sigma}_{22}(t) = \dot{\sigma}_{33}(t) &= \delta(0)\sigma_1\frac{\nu}{1-\nu} - \dot{\varepsilon}_{22}(t)\frac{E}{1-\nu}.\end{aligned}$$

Growth speed tensor is given by (Eq. 1):

$$\dot{\varepsilon}_{11}(t) = \dot{\varepsilon}_B + \dot{\varepsilon}_B\int_0^t [\kappa_1 e^{-\frac{t-\tau}{\tau_1}}(\dot{\sigma}_{11} + 2\dot{\sigma}_{22}) + (\gamma_0 + \gamma_1 e^{-\frac{t-\tau}{\nu_1}})\dot{\sigma}_{11}]\mathrm{d}\tau \tag{5}$$

$$= \dot{\varepsilon}_B + \dot{\varepsilon}_B[\kappa_1 e^{-\frac{t}{\tau_1}}\sigma_1(1 + \frac{2\nu}{1-\nu}) + (\gamma_0 + \gamma_1 e^{-\frac{t}{\nu_1}})\sigma_1 - \frac{2\kappa_1 E}{1-\nu}\int_0^t e^{-\frac{t-\tau}{\tau_1}}\dot{\varepsilon}_{22}(\tau)\mathrm{d}\tau] \tag{6}$$

$$= \dot{\varepsilon}_B + \dot{\varepsilon}_B\{[\kappa_1(1 + \frac{2\nu}{1-\nu})e^{-\frac{t}{\tau_1}} + \gamma_0 + \gamma_1 e^{-\frac{t}{\nu_1}}]\sigma_1 - \frac{2\kappa_1 E}{1-\nu}\int_0^t e^{-\frac{t-\tau}{\tau_1}}\dot{\varepsilon}_{22}(\tau)\mathrm{d}\tau\} \tag{7}$$

$$\dot{\varepsilon}_{22}(t) = \dot{\varepsilon}_B\int_0^t [\kappa_1 e^{-\frac{t-\tau}{\tau_1}}(\dot{\sigma}_{11} + 2\dot{\sigma}_{22}) + (\gamma_0 + \gamma_1 e^{-\frac{t-\tau}{\nu_1}})\dot{\sigma}_{22}]\mathrm{d}\tau \tag{8}$$

$$= \dot{\varepsilon}_B\{\sigma_1[\kappa_1 e^{-\frac{t}{\tau_1}}(1 + \frac{2\nu}{1-\nu}) + (\gamma_0 + \gamma_1 e^{-\frac{t}{\nu_1}})\frac{\nu}{1-\nu}] \tag{9}$$

$$- \frac{E}{1-\nu}\int_0^t (2\kappa_1 e^{-\frac{t-\tau}{\tau_1}} + \gamma_0 + \gamma_1 e^{-\frac{t-\tau}{\nu_1}})\dot{\varepsilon}_{22}(\tau)\mathrm{d}\tau\} \tag{10}$$

$$\dot{\varepsilon}_{33}(t) = \dot{\varepsilon}_{22}(t) \tag{11}$$

It was not possible to obtain analytical solutions for these equations. Nevertheless, it is possible to compute the asymptotic values of growth speeds by applying the final value theorem of the Laplace transform. By applying a Laplace transform to both sides of Eq. 9 we obtain:

$$\mathscr{L}(\dot{\varepsilon}_{22}) = \dot{\varepsilon}_B\{\sigma_1[\kappa_1(1 + \frac{2\nu}{1-\nu})\frac{1}{s+\frac{1}{\tau_1}} + \frac{\nu}{1-\nu}(\frac{\gamma_0}{s} + \gamma_1\frac{1}{s+\frac{1}{\nu_1}})] - \frac{E}{1-\nu}(\kappa_1\frac{2}{s+\frac{1}{\tau_1}} + \frac{\gamma_0}{s} + \gamma_1\frac{1}{s+\frac{1}{\nu_1}})\mathscr{L}(\dot{\varepsilon}_{22})\}$$

from where it follows that:

$$\begin{aligned}\mathscr{L}(\dot{\varepsilon}_{22}) &= \frac{\dot{\varepsilon}_B\sigma_1[\kappa_1(1+\frac{2\nu}{1-\nu})\frac{1}{s+\frac{1}{\tau_1}} + \frac{\nu}{1-\nu}(\frac{\gamma_0}{s} + \frac{1}{2}\gamma_1\frac{1}{s+\frac{1}{\nu_1}})]}{1 + \dot{\varepsilon}_B\frac{2E}{1-\nu}(\kappa_1\frac{1}{s+\frac{1}{\tau_1}} + \frac{\frac{1}{2}\gamma_0}{s} + \frac{1}{2}\gamma_1\frac{1}{s+\frac{1}{\nu_1}})}\\ \lim_{s\to 0} s\mathscr{L}(\dot{\varepsilon}_{22}) &= 0\\ &= \lim_{t\to\infty}\dot{\varepsilon}_{22}(t).\end{aligned}$$

This result implies that the solution for long times is such that there is no growth in directions 2 and 3, and a stable value of stress is reached. Similarly, for $\dot{\varepsilon}_{11}(t)$ we obtain:

$$\begin{aligned}\mathscr{L}(\dot{\varepsilon}_{11}) &= \frac{\dot{\varepsilon}_B}{s} + \dot{\varepsilon}_B[\frac{\kappa_1\sigma_1}{s+\frac{1}{\tau_1}}(1+\frac{2\nu}{1-\nu}) + (\frac{\gamma_0}{s} + \frac{\gamma_1}{s+\frac{1}{\nu_1}})\sigma_1 - \frac{2\kappa_1 E}{1-\nu}\frac{\mathscr{L}(\dot{\varepsilon}_{22})}{s+\frac{1}{\tau_1}}] \\ \lim_{s\to 0} s\mathscr{L}(\dot{\varepsilon}_{11}) &= \dot{\varepsilon}_B + \dot{\varepsilon}_B\gamma_0\sigma_1.\end{aligned}$$

Similarly, growth plate epiphyseal height is given by:

$$\begin{aligned}\frac{\dot{\mathsf{e}}}{\mathsf{e}} &= \dot{\varepsilon}_{11} - \mathrm{tr}(\dot{\varepsilon}) \\ &= -2\dot{\varepsilon}_{22} \\ \ln[\frac{\mathsf{e}(t\to\infty)}{\mathsf{e}(0)}] &= \lim_{t\to\infty} -2\int_0^t \dot{\varepsilon}_{22}(\tau)\mathrm{d}\tau \\ &= \lim_{s\to 0} s[2\frac{1}{s}\mathscr{L}(\dot{\varepsilon}_{22})] \\ &= \lim_{s\to 0} -2\frac{\dot{\varepsilon}_B\sigma_1[\kappa_1(1+\frac{2\nu}{1-\nu})\frac{1}{s+\frac{1}{\tau_1}} + \frac{\nu}{1-\nu}(\frac{\gamma_0}{s} + \gamma_1\frac{1}{s+\frac{1}{\nu_1}})]}{1+\dot{\varepsilon}_B\frac{2E}{1-\nu}(\kappa_1\frac{1}{s+\frac{1}{\tau_1}} + \frac{1}{2}\frac{\gamma_0}{s} + \frac{1}{2}\gamma_1\frac{1}{s+\frac{1}{\nu_1}})} \\ &= -2\sigma_1\frac{\nu}{E}\end{aligned}$$

thus,

$$\mathsf{e}(t\to\infty) = \mathsf{e}e^{-2\sigma_1\frac{\nu}{E}}.$$

Following these results, as analytical solutions are not available, the code was verified by checking that, asymptotically, epiphyseal plate height is not divergent, and corresponding growth speeds are consistent with the model equations, Fig. 1. For this particular case, asymptotic growth speed converges to the analytical solution within the machine error. This case implementation can be found in the provided examples, as "rheologycal_2D_validation.dgibi".

2.2 Torsional loads

Solution of a prototypical case of growth for a cylindrical geometry was implemented, Fig. 2(a), and compared against previously obtained analytical solutions [1]. For a cylindrical bone of radius r and epiphyseal thickness e, in which a sudden torque T is applied, solution for induced angular deviation can be analytically found, being:

$$\dot{\theta}(t) = \frac{eT\dot{\varepsilon}_B}{\frac{\pi r^3}{2}}[\gamma + \gamma_1\tau_1(e^{-\frac{t}{\nu_1}} - 1)].$$

This case implementation is provided in the file "growth1_rheo_tors.dgibi", along the software implementation examples. Notice that, in this case, elements distortion increases as torsional rotation occurs, so in order to obtain precise solutions for long simulation times, remeshing should be applied. For brevity, convergence rate of solution is not shown here. Solution error in the showed case is within 1%, Fig. 2(b).

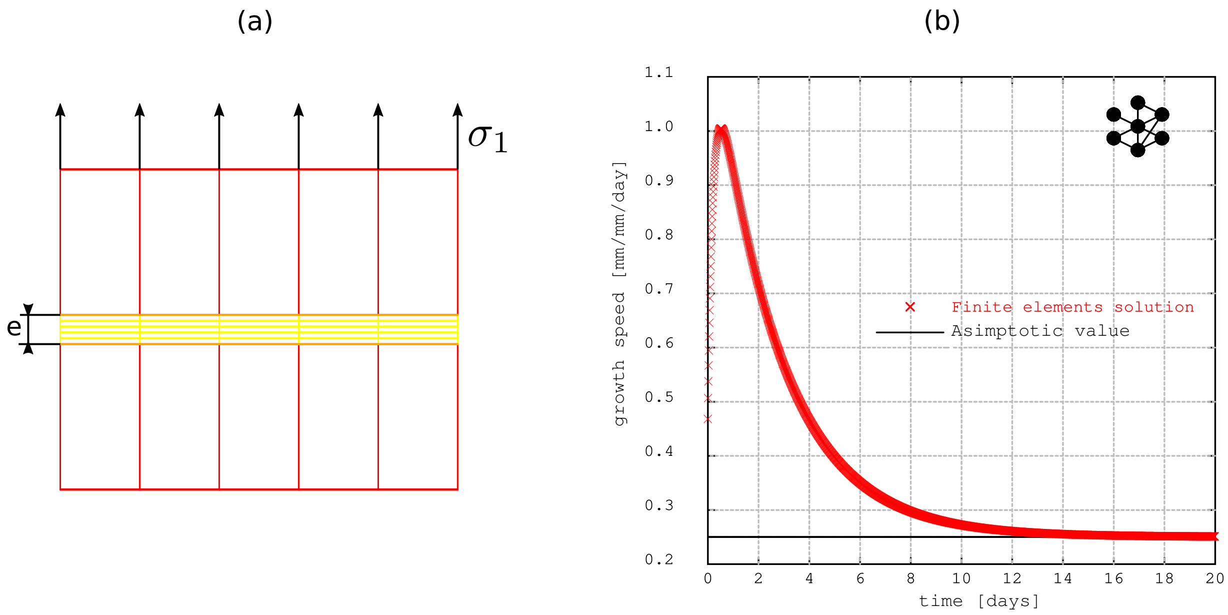

Figure 1: Geometry and growth predicted speed for verification case 1. Errors in predicted growth speed asymptotically reduce to the machine error for this case.

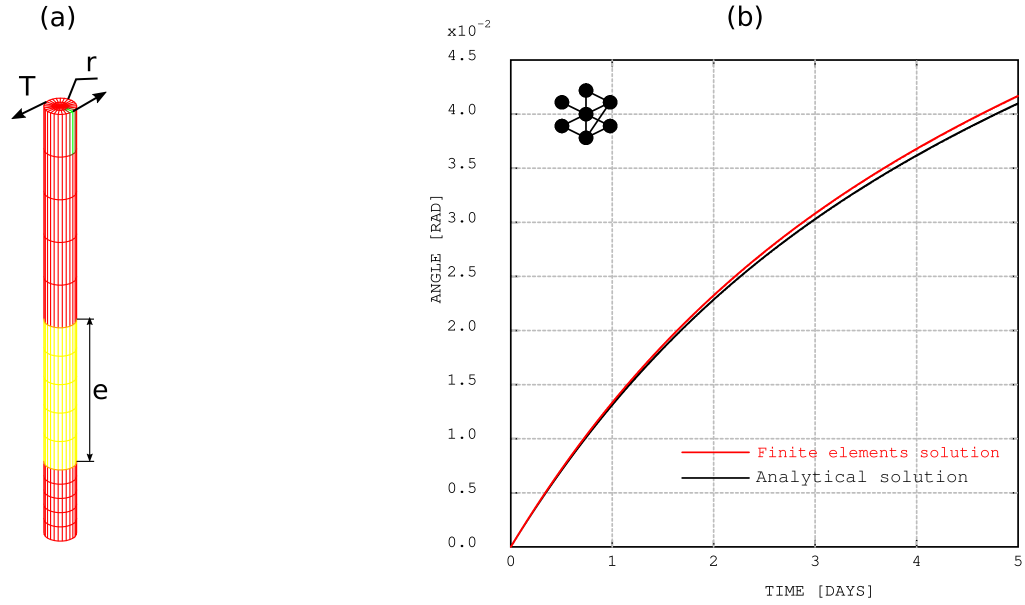

Figure 2: Verification case of the numerical solution obtained for a prototypical torsional case.

References

[1] M. G. Alonso, A. Yawny, and G. Bertolino. How do bones grow? a mathematical description of the mechanobiological behavior of the epiphyseal plate. *Biomechanics and Modeling in Mechanobiology*, pages 1617–7940, June 2022. doi: 10.1007/s10237-022-01608-y. URL https://doi.org/10.1007/s10237-022-01608-y.

[2] M.G. Alonso, A. Yawny, and G. Bertolino. A tool for solving bone growth related problems using finite elements adaptive meshes. *Journal of the Mechanical Behavior of Biomedical Materials*, page 104946, November 2021. doi: 10.1016/j.jmbbm.2021.104946. URL https://doi.org/10.1016/j.jmbbm.2021.104946.
