# Supplementary material for: A numerical study towards shape memory alloys application in orthotic management of pediatric knee lateral deviations
Source: Sci Rep. 2023 Feb 6;13:2134. doi: 10.1038/s41598-023-29254-z (PMC9902535; doi:10.1038/s41598-023-29254-z)
Supplement: Supplementary file 1 — Supplementary Information. [file 41598_2023_29254_MOESM1_ESM.zip › Sup_mats/Sup_Fig_2.pdf]

Estimated relative error on computed angle after 60 days simulation period.

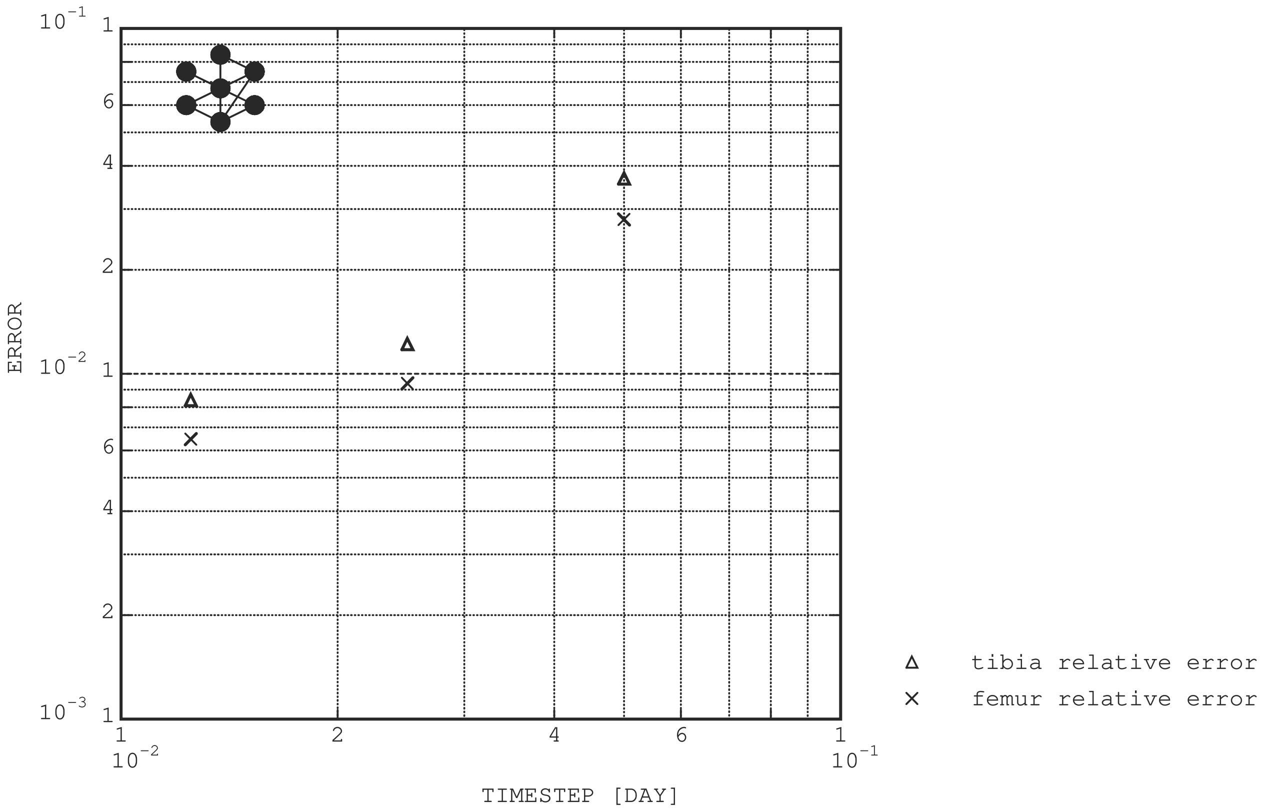

Solutions were compared against the obtained for dt = 0.001 day.
