# Supplementary material for: A numerical study towards shape memory alloys application in orthotic management of pediatric knee lateral deviations
Source: Sci Rep. 2023 Feb 6;13:2134. doi: 10.1038/s41598-023-29254-z (PMC9902535; doi:10.1038/s41598-023-29254-z)

Uncertainty estimation of maximum applicable correcting flexor moment.

Results were obtained after 100 stochastic computations of the maximum applicable flexor moment.
Values were obtained for an admisible stress of 0.153 MPa.

Max. flexor moment based on the von Mises stress:

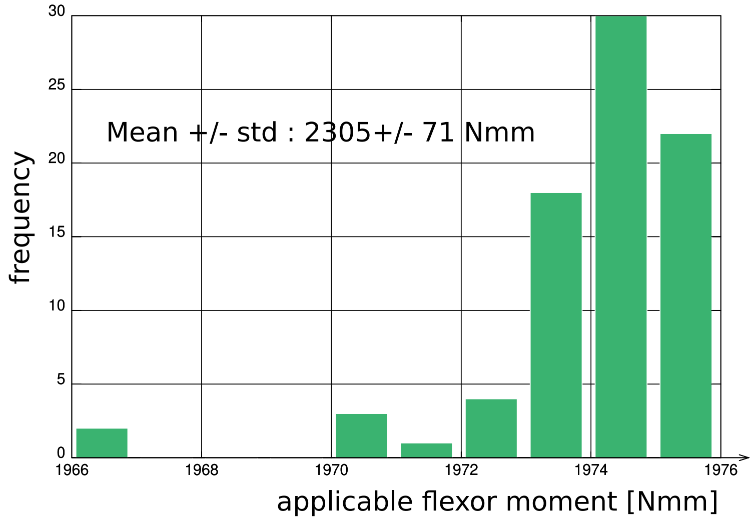

Max. flexor moment based on the Tresca stress:

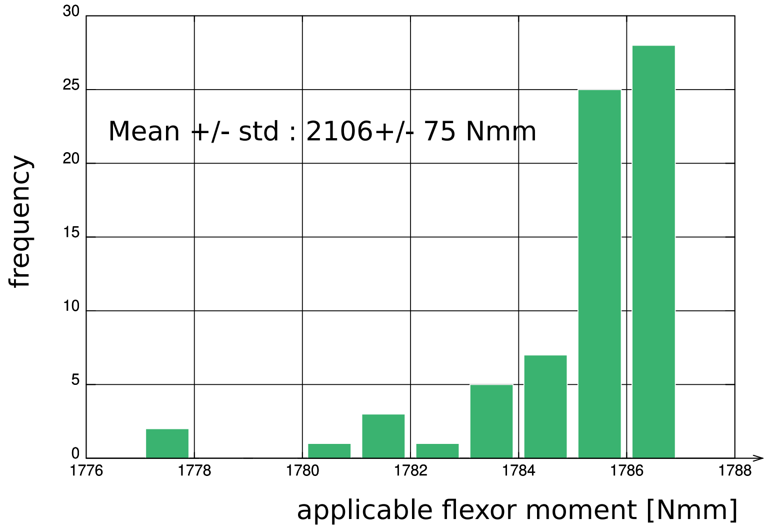

Max. flexor moment based on the max. principal stress:

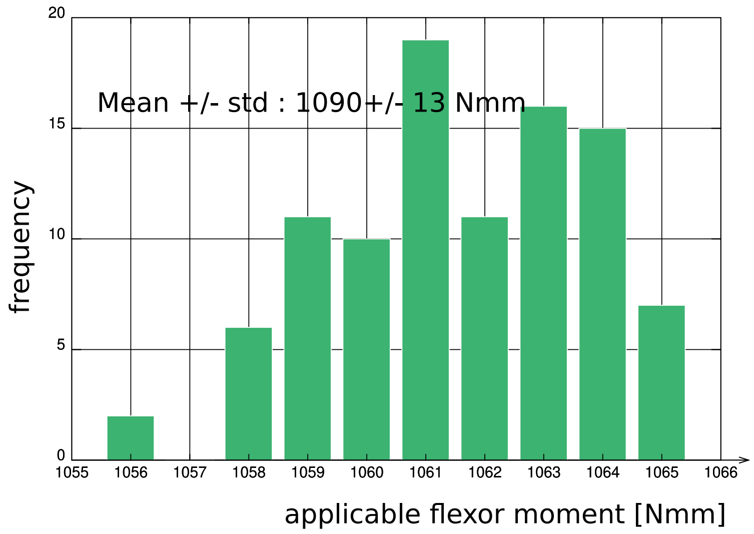

Supplement: Supplementary file 1 — Supplementary Information. [file 41598_2023_29254_MOESM1_ESM.zip › Sup_mats/Sup_Fig_4.pdf]
