# Supplementary material for: A numerical study towards shape memory alloys application in orthotic management of pediatric knee lateral deviations
Source: Sci Rep. 2023 Feb 6;13:2134. doi: 10.1038/s41598-023-29254-z (PMC9902535; doi:10.1038/s41598-023-29254-z)
Supplement: Supplementary file 1 — Supplementary Information. [file 41598_2023_29254_MOESM1_ESM.zip › Sup_mats/Sup_Tab_1.pdf]

Estimated typical efforts on the knee modeled tissues for different activities.

Knee segment	Criteria	Applied load			
		standing (0.5BW)	cycling (1.25BW)	walking (2.8BW)	jogging (4.2BW)
Femoral cartilage	VM stress [MPa]	5.54(1.9)	5.65(1.7)	5.94(1.8)	6.28(1.7)
	cont. press. [MPa]	1.92(3.4)	1.99(3.5)	2.15(3.0)	2.28(3.0)
Med. tib. cart.	VM stress [MPa]	8.42(3.6)	8.63(1.9)	9.78(2.3)	9.33(2.4)
	cont. press. [MPa]	5.29(1.9)	5.49(1.4)	5.91(2.1)	6.30(5.2)
Lat. tib. cart.	VM stress [MPa]	5.07(2.4)	5.15(2.2)	5.29(2.0)	5.40(1.8)
	cont. press. [MPa]	4.96(1.5)	5.14(1.4)	5.51(1.2)	5.89(1.8)
Medial meniscus	VM stress [MPa]	5.77(3.0)	5.92(2.3)	6.26(2.4)	6.59(2.8)
	cont. press. [MPa]	1.94(7.0)	1.90(6.4)	1.89(5.2)	1.85(2.4)
	deflection [mm]	1.39(<0.5)	1.43(<0.5)	1.49(<0.5)	1.56(<0.5)
Lateral meniscus	VM stress [MPa]	10.9(2.3)	10.8(3.3)	11.5(3.2)	12.23(3.2)
	cont. press. [MPa]	3.48(5.8)	3.54(6.0)	3.68(1.9)	3.93(2.3)
	deflection [mm]	1.51(<0.5)	1.54(<0.5)	1.61(<0.5)	1.68(<0.5)

(Numbers between parenthesis indicate the local estimated solution accuracy as percent error.)

Estimated typical efforts on the knee modeled tissues for different applied flexor moments.

Knee segment	Criteria	Applied flexor moment							
		3 Nm	6 Nm	9 Nm	12 Nm	15 Nm	18 Nm	21 Nm	24 Nm
Femoral cartilage	VM stress [MPa]	1.94(2.6)	2.64(2.4)	2.94(2.0)	3.10(1.7)	3.34(1.7)	3.56(1.6)	3.71(1.4)	3.85(1.4)
	cont. press. [MPa]	0.15(3.7)	0.27(6.7)	0.39(8.0)	0.46(2.7)	0.53(2.1)	0.64(1.5)	0.71(2.8)	0.78(4.5)
Med. tib. cart.	VM stress [MPa]	0.59(5.0)	0.50(5.4)	1.54(9.2)	1.85(2.58)	1.95(5.9)	2.20(6.5)	2.63(7.7)	2.82(5.4)
	cont. press. [MPa]	0.14(9.6)	0.12(9.2)	0.35(5.2)	0.63(3.6)	0.84(4.7)	0.95(0.5)	1.02(0.4)	1.12(0.5)
Lat. tib. cart.	VM stress [MPa]	3.51(4.1)	3.99(4.1)	4.26(4.0)	4.53(4.1)	4.80(4.0)	4.93(3.6)	5.12(3.2)	5.29(2.4)
	cont. press. [MPa]	1.04(6.3)	1.17(1.3)	1.86(2.0)	2.26(3.2)	2.60(7.5)	2.80(2.6)	2.69(0.7)	2.97(9.4)
Medial meniscus	VM stress [MPa]	1.50(2.8)	1.62(2.9)	1.30(2.2)	1.39(2.9)	1.44(2.7)	1.56(2.5)	1.84(2.4)	1.93(2.5)
	cont. press. [MPa]	0.33(7.7)	0.35(5.7)	0.29(0.5)	0.30(5.0)	0.30(4.5)	0.33(1.7)	0.36(4.4)	0.38(6.4)
	deflection [mm]	0.93(<0.5)	0.90(<0.5)	0.91(<0.5)	0.91(<0.5)	0.92(<0.5)	0.93(<0.5)	0.93(<0.5)	0.94(<0.5)
Lateral meniscus	VM stress [MPa]	5.00(2.5)	5.08(2.6)	6.99(5.8)	9.62(1.8)	11.33(1.9)	11.39(1.9)	10.83(1.7)	9.95(1.4)
	cont. press. [MPa]	1.42(6.4)	2.07(4.9)	2.34(6.1)	2.76(7.7)	3.11(7.0)	3.46(7.5)	3.70(7.4)	3.87(6.7)
	deflection [mm]	0.83(<0.5)	0.84(<0.5)	0.94(<0.5)	1.14(<0.5)	1.31(<0.5)	1.48(<0.5)	1.63(<0.5)	1.77(<0.5)

(Numbers between parenthesis indicate the local estimated solution accuracy as percent error.)
