# Supplementary material for: A numerical study towards shape memory alloys application in orthotic management of pediatric knee lateral deviations
Source: Sci Rep. 2023 Feb 6;13:2134. doi: 10.1038/s41598-023-29254-z (PMC9902535; doi:10.1038/s41598-023-29254-z)
Supplement: Supplementary file 1 — Supplementary Information. [file 41598_2023_29254_MOESM1_ESM.zip › Sup_mats/Sup_Mat_8.pdf]

Solution convergence for loads up to 4.8 BW.

Mesh refinements:

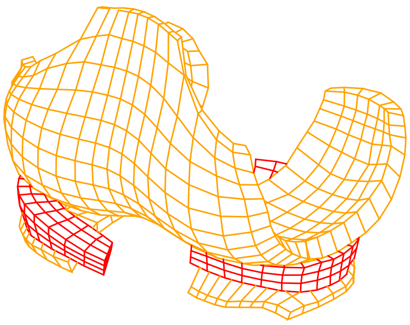

Coarse mesh H

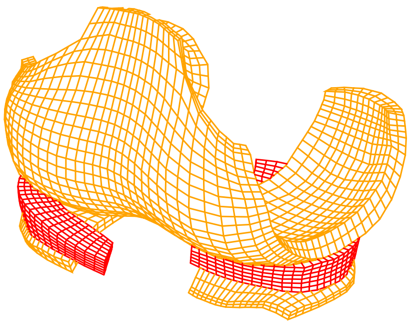

Medium mesh H/2

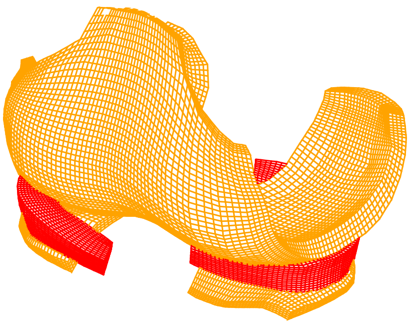

Fine mesh H/4

Global error on computed displacements

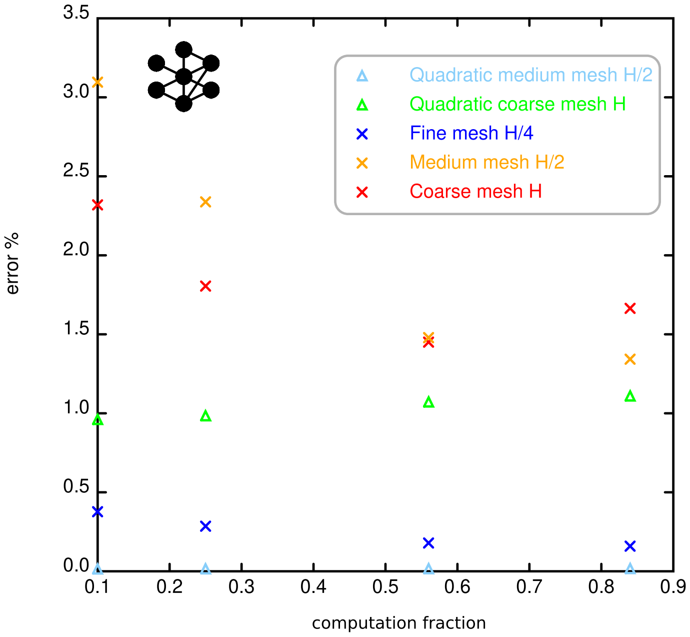

Global error was computed as:

$$\varepsilon_{\%} = \max\left(\sqrt{\int_{\Omega} \frac{(u_i - u_i^0)}{u_i^0} dV}\right) \times 100\%$$

having estimated u_i^0 from the Richardson extrapolation of the three higher refinement linear meshes solutions.

Global error on computed stresses

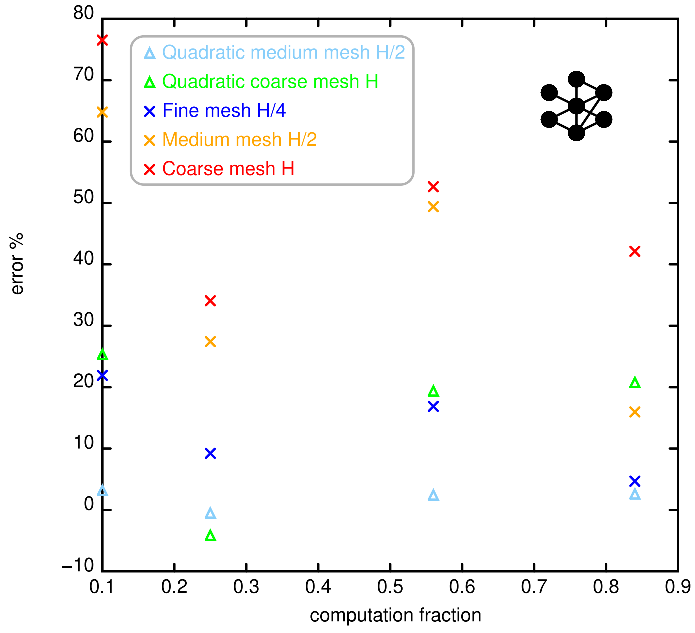

Global error was computed as:

$$\varepsilon_{\%} = \max\left(\sqrt{\int_{\Omega} \frac{(\sigma_{ij} - \sigma_{ij}^0)}{\sigma_{ij}^0} dV}\right) \times 100\%$$

having estimated σ_{ij}^0 from the Richardson extrapolation of the three higher refinement linear meshes solutions.

Tension in the considered ligament bundles for loads up to 4.8 Bw

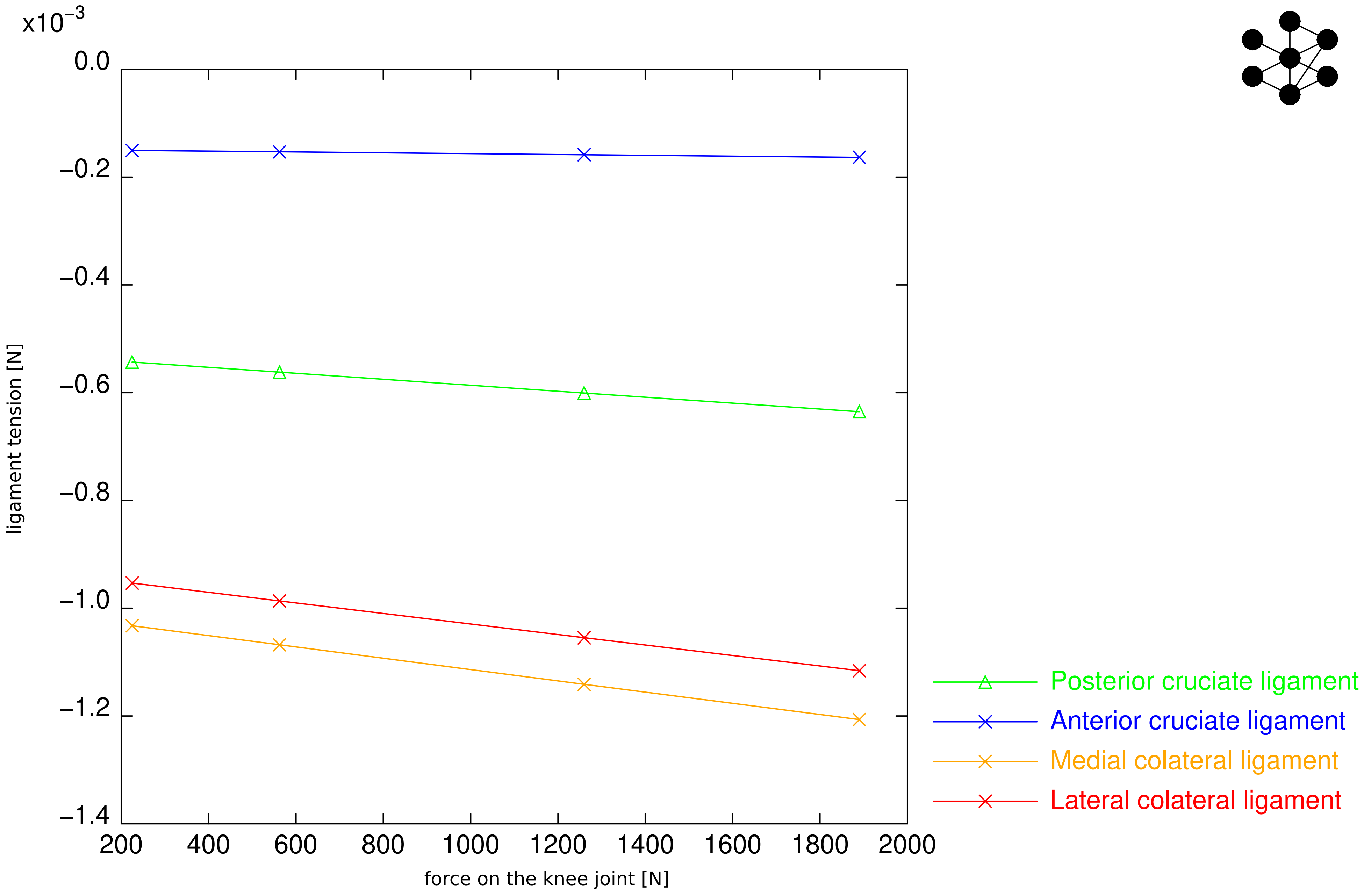

Solution convergence for flexor moments up to 90 Nm.

Mesh refinements:

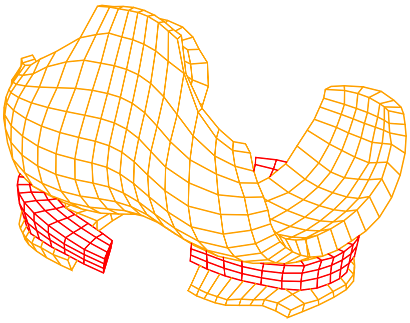

Coarse mesh H

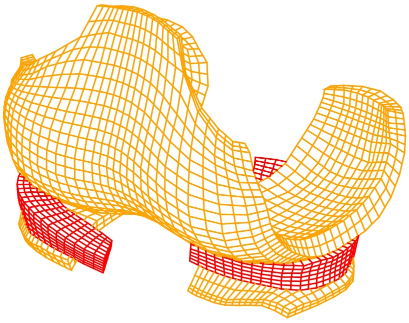

Medium mesh H/2

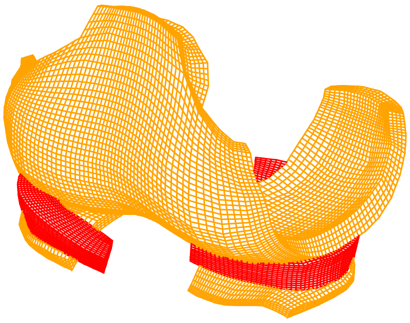

Fine mesh H/4

Global error on computed displacements

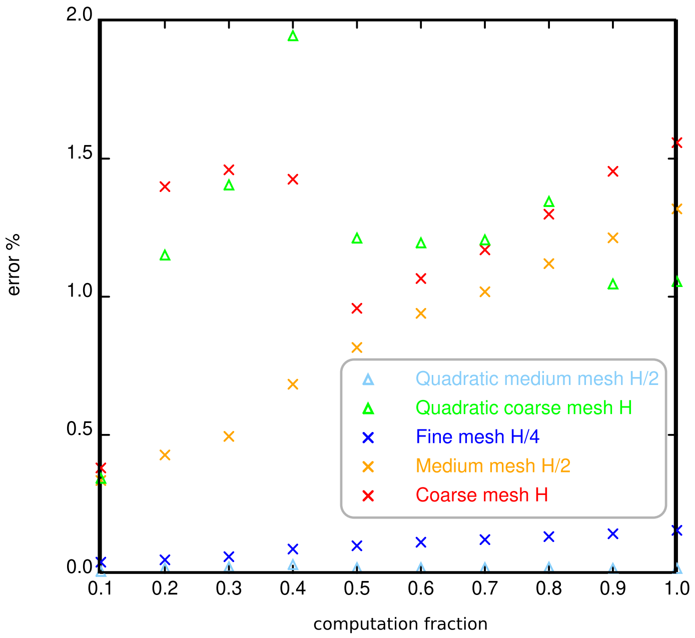

Global error was computed as:

$$\varepsilon_{\%} = \max\left(\sqrt{\int_{\Omega} \frac{(u_i - u_i^0)}{u_i^0} dV}\right) \times 100\%$$

having estimated u_i^0 from the Richardson extrapolation of the three higher refinement linear meshes solutions.

Global error on computed stresses

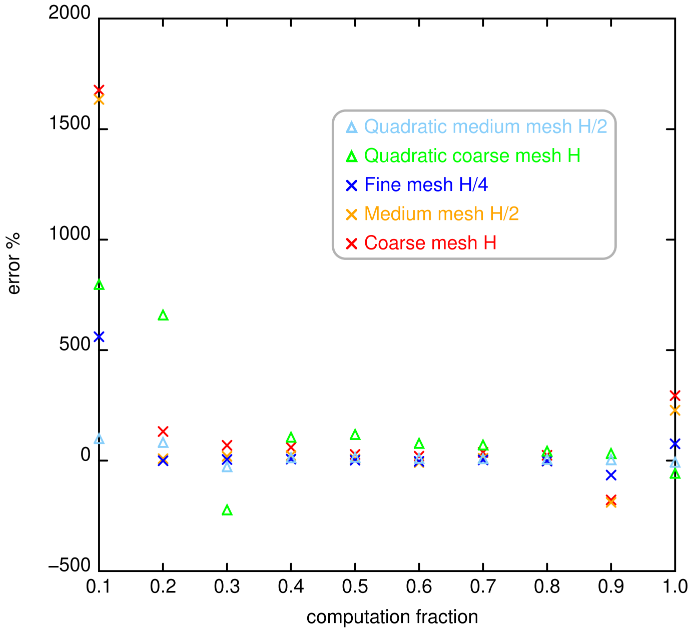

Global error was computed as:

$$\varepsilon_{\%} = \max\left(\sqrt{\int_{\Omega} \frac{(\sigma_{ij} - \sigma_{ij}^0)}{\sigma_{ij}^0} dV}\right) \times 100\%$$

having estimated σ_{ij}^0 from the Richardson extrapolation of the three higher refinement linear meshes solutions.

Tension in the considered ligament bundles for a flexor moment up to 90 Nm.

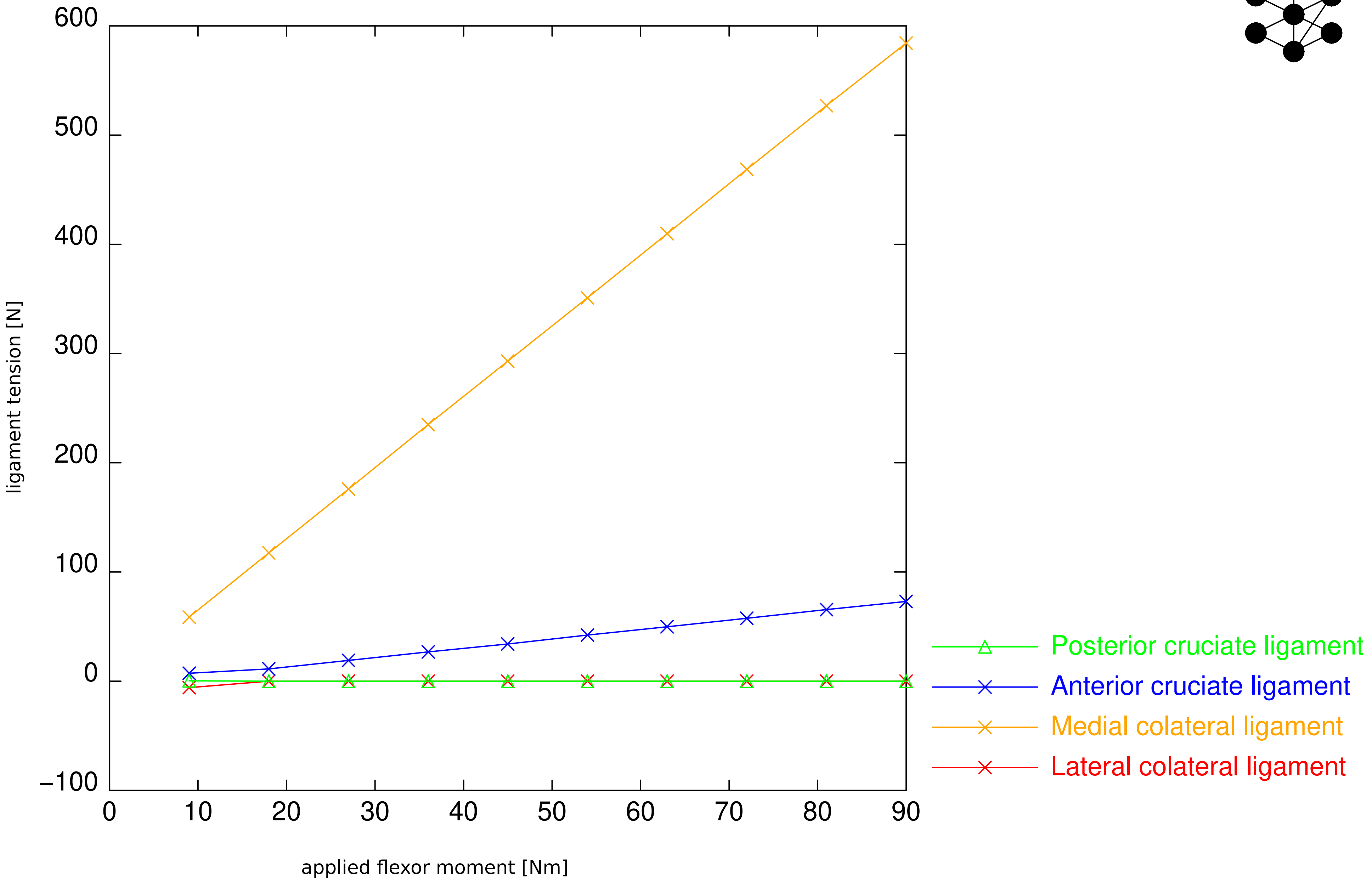

LOADS ON THE LIGAMENTS
