# Supplementary figures and images for: A numerical study towards shape memory alloys application in orthotic management of pediatric knee lateral deviations
Source: Sci Rep. 2023 Feb 6;13:2134. doi: 10.1038/s41598-023-29254-z (PMC9902535; doi:10.1038/s41598-023-29254-z)

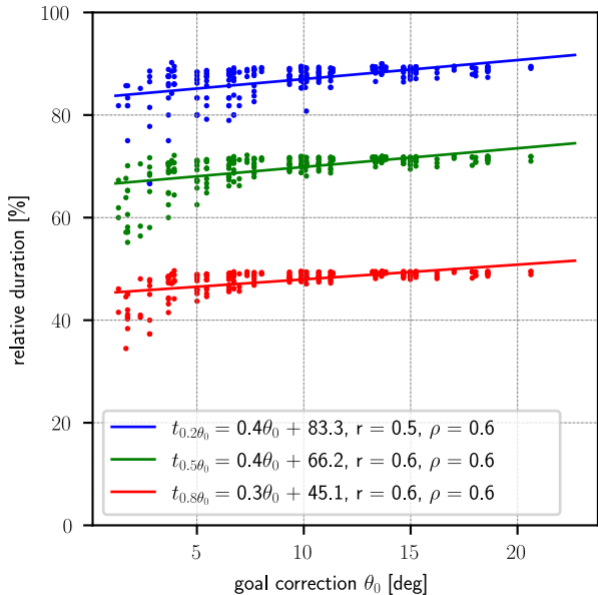

Supplement: Supplementary file 1 — Supplementary Information. [file 41598_2023_29254_MOESM1_ESM.zip › Sup_mats/Sup_Fig_6.pdf]

(a)

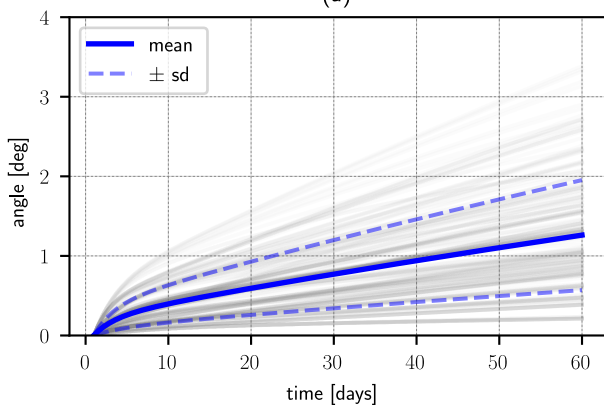

(b)

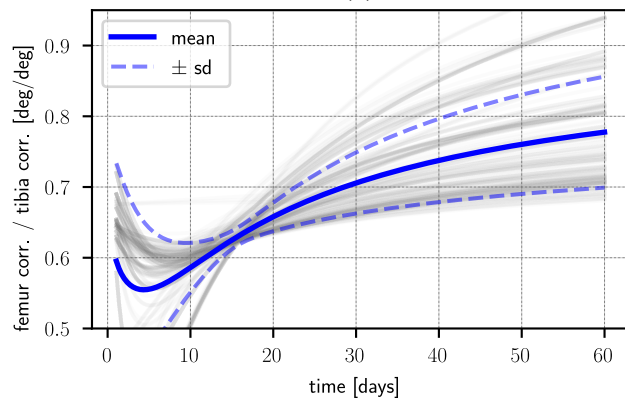

(c)

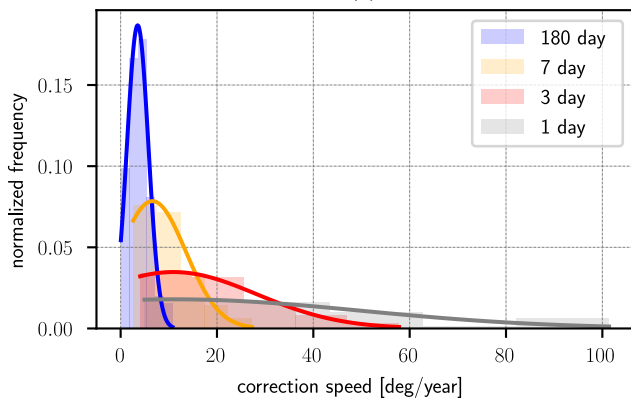

(d)

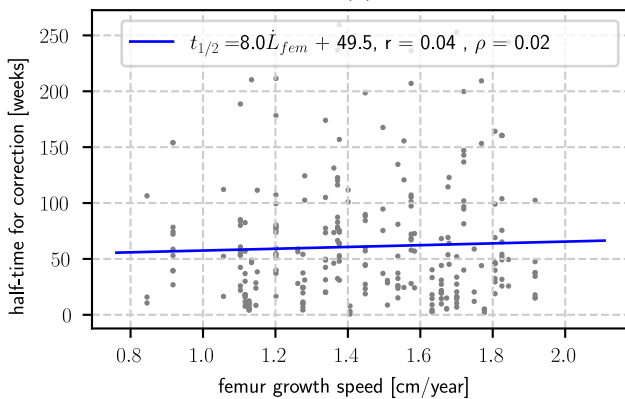

(e)

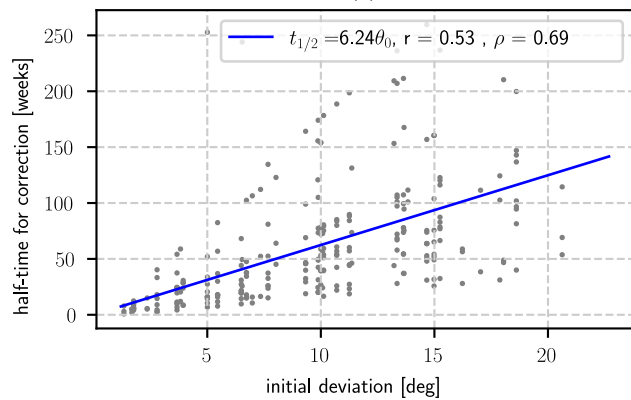

(f)

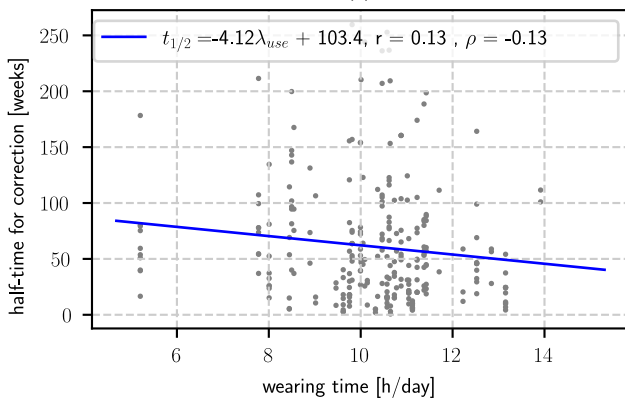

Supplement: Supplementary file 1 — Supplementary Information. [file 41598_2023_29254_MOESM1_ESM.zip › Sup_mats/Sup_Fig_5.pdf]

(a)

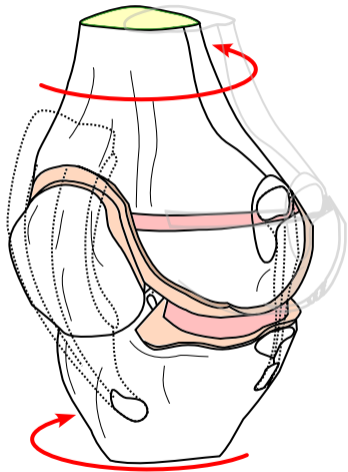

(b)

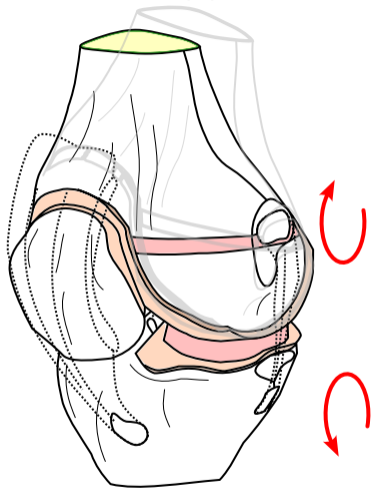

Supplement: Supplementary file 1 — Supplementary Information. [file 41598_2023_29254_MOESM1_ESM.zip › Sup_mats/Sup_Fig_7.pdf]
